# Comparative efficacy and tolerability of targeted and immunotherapy combined with chemotherapy as first-line treatment for advanced gastric cancer: a Bayesian network meta-analysis

**DOI:** 10.1038/s41598-022-24426-9

**Published:** 2022-12-20

**Authors:** Shu Liu, Heung Yan Wong, Li Xie, Yoojin Kim, Danhua Shu, Beishi Zheng, Naxin Liu, Chungen Xing, Xiaolei Chen, Qiantong Dong

**Affiliations:** 1 Department of Biomedical Sciences, City University of Hong Kong, Kowloon Tong, Hong Kong; 2 Jockey Club College of Veterinary Medicine and Life Sciences, City University of Hong Kong, Kowloon Tong, Hong Kong; 3 School of Biomedical Sciences, Faculty of Health, Queensland University of Technology, Brisbane, QLD Australia; 4 Internal Medicine Department, Woodhull Medical and Mental Health Center, Brooklyn, NY USA; 5 Department of Gastrointestinal Surgery, The First Affiliated Hospital of Wenzhou Medical University, Wenzhou, China; 6 Department of General Surgery, The Second Affiliated Hospital of Soochow University, Suzhou, Jiangsu Province China

**Keywords:** Cancer therapy, Gastrointestinal cancer, Gastroenterology

## Abstract

The use of target agents and immune checkpoint inhibitors have changed the treatment landscape for AGC in the first-line setting. However, the crosswise comparison between each regimen is rare. Therefore, we estimated the efficacy and safety of targeted therapy or immunotherapy with chemotherapy in AGC patients as the first-line treatment. Included studies were divided into “average” or “specific positivity” group according to whether the patients were selected by a certain pathological expression. We conducted a Bayesian network meta-analysis for all regimens in both groups. In average group, no regimen showed significant improvements in overall survival (OS) and progression free survival (PFS), while pembrolizumab and nivolumab combined with chemotherapy were ranked first and second respectively without an obvious safety difference. In specific positivity group, zolbetuximab plus chemotherapy significantly prolonged OS (HR 0.53, 95% CI 0.36–0.79) and PFS (HR 0.45, 95% CI 0.25–0.81). The top three regimens were zolbetuximab-chemotherapy, trastuzumab plus pertuzuma-chemotherapy and nivolumab-chemotherapy respectively, with no significant safety risk. For average patients, immune checkpoint inhibitor PD-1 plus chemotherapy will be the promising regimen. For patients with overexpression of CLDN18.2, zolbetuximab combined with chemotherapy comes with greater survival benefits, while for patients who have PD-L1 expression with no HER-2 or CLDN18.2 positivity, additional immune checkpoint inhibitor of PD-1 will be a good considered option.

## Introduction

Gastric cancer was the fifth most commonly diagnosed cancer and the fourth leading cause of cancer death in 2020, with an especially high incidence in Eastern Asia^1^. It was estimated that over one million new cases occurred in 2020 with 769,000 reported deaths, which illustrates its relatively poor prognosis^2^. The reason for the high mortality in gastric cancer patients is attributed to the fact that approximately 50% of the patients are presented for late-stage diagnoses.

For early-stage gastric cancer patients, curative surgical resection is recommended as the optimal therapeutic option^3^. However, in the case of advanced gastric cancer (AGC) that is unresectable, metastatic, recurring, or locally advanced, systemic therapies including chemotherapy, targeted therapy, immunotherapy, and their combined regimens are often used as preferred palliative treatments, which not only offer survival benefits but also increase the chances for the next curative surgery.

Recently, great progress has been made in first-line regimens for untreated AGC. Firstly, double or triple platinum-fluoropyrimidine combinations have become the standard first-line chemotherapy in the National Comprehensive Cancer Network (NCCN) clinical practice guidelines^4^. Secondly, trastuzumab, a target agent against human epidermal growth factor receptor II (HER-2), was recommended as an additional targeted therapy combined with first-line chemotherapy for HER-2 positive patients. Furthermore, based on successful results from CheckMate-649 study^5^, nivolumab, an anti-programmed cell death protein 1 (PD-1) antibody, combined with chemotherapy has become the new standard first-line treatment in NCCN guidelines for AGC among patients whose programmed death-ligand 1 (PD-L1) combined positive score (CPS) is 5 or higher while HER-2 overexpression is negative.

Since 2010, large number of randomized controlled trials (RCTs) have explored the efficacy and safety of different targeted therapies or immunotherapies and compared them with standard chemotherapy as first-line treatment among AGC patients. Although these research progresses are likely to change the landscape of first-line treatments, comparisons between different regimens are still lacking, especially evaluation between targeted therapy and immunotherapy. Network meta-analyses can evaluate and rank the effects of various treatments via direct or indirect evidence, which provides an ideal approach to the field of cancer research. Although Cheng et al. summarized first-line systemic therapies for AGC in 2019 by network meta-analysis, all target medications were combined into one node rather than evaluating the efficacy and establishing ranks between them^6^. In 2017, Xie et al. published a comparison of target agents used in combination with chemotherapy in untreated AGC patients^7^, but this study erroneously mixed several second-line therapy RCTs. Furthermore, neither study included any immunotherapy trials owing to their early publication when the evaluation of targeted therapy and immunotherapy was still lacking.

In this study, we conducted a Bayesian network meta-analysis to evaluate and rank the efficacy and tolerability of target agents or immune checkpoint inhibitors combined with standard chemotherapy as first-line treatment in untreated AGC patients, which will help in clinical decision-making for future patients receiving first-line AGC therapy.

## Materials and methods

### Search strategy

PubMed, Cochrane Central Register of Controlled Trials databases and Embase database were searched for studies published before August 25, 2021.We used relevant combinations, keywords and MeSH (Medical Subject Heading) terms pertaining to disease (e.g., gastric cancer, stomach neoplasm, esophagogastric cancer), therapy (e.g., chemotherapy, immunotherapy, targeted-therapy), disease stage (e.g., advanced, unresected, metastatic). Furthermore, several previously published high-quality systematic reviews were also reviewed in case of omission. Full electronic search strategy is shown in the supplementary material (Supplement Table S1).

### Selection criteria

Under the PICOS framework, studies were considered eligible when they met all of the following inclusion criteria: Participant: patients bore untreated AGC, including locally inoperable or unresectable, advanced, recurrent, and metastatic cases. Studies containing lower esophageal cancer cases were eligible. Studies whose patients received the last adjuvant chemotherapy more than 6 months past were also eligible, but studies without a clear indication of the time of the last adjuvant chemotherapy were not included; Intervention: different target agents or immune checkpoint inhibitors in combination with standard first-line chemotherapy against AGC. We only included studies in which chemotherapy was the first-line regimen in accordance with NCCN 2020 guidelines for AGC. Otherwise, studies were not qualified; Comparator: chemotherapy with or without placebo compared with chemotherapy plus different target agents or immune checkpoint inhibitors; Outcome: overall survival (OS) and progression free survival (PFS) are primary outcomes, while objective response rate (ORR) and adverse events (AE) are secondary outcomes; Study design: phase II and phase III randomized controlled trials reported before August 2021 without language limitation. When one registered trial had several different reports, we only included the one with the longest follow-up rather than the subgroup report.

Studies were excluded if they met at least one of the following exclusion criteria: Comparison between each arm cannot be incorporated into network calculation; Chemotherapy regimens are not qualified with first-line chemotherapy standard; Patients in studies had received their last adjuvant chemotherapy within 6 months, or the precise time of the last adjuvant chemotherapy is not reported.

The protocol of our systematic review and network meta-analysis had been published in PROSPERO (CRD42021271480).

### Data extraction and risk of bias assessment

The following information in studies has been extracted by two authors independently. 1. General characteristics of the studies: name of the first-author, publication year, and the national clinical trial (NCT) registration number. 2. Patient baseline characteristics: age, region, follow-up time, number of peritoneal metastases, tumor location, and whether they had any specific pathological positivity. 3. Treatment in different arms: the regimens of chemotherapy, target agents or immune checkpoint inhibitors, and the sample size in each treatment. 4. Primary and secondary outcomes: OS, PFS, presented with hazard ratios (HRs) and 95% confidence intervals (95% CIs). Risk ratios (RR) and 95% CIs were applied as the effect size for ORR, AE ≥ 3. ORR was defined as the proportion of patients who reached a partial or complete response. AE ≥ 3 means only Grade 3 or higher adverse events were counted, following the National Cancer Institute Common Terminology Criteria for Adverse Events (CTCAE). When HRs and 95% CIs were not directly provided, we used Engauge Digitizer 4.0 to extract survival probabilities at different timepoints. After a series survival probability-time point prepared, spreadsheet created by Tierney JF^8^ was used to generated mimic survival curve then estimated the HR, 95% CIs. For the multiple-arm trial, the variance of baseline treatment (Chemotherapy) was estimated by David Scott^9^ method. The risk of bias in each included study was assessed by Cochrane Collaboration tool^10^, which assigns grades of “high risk”, “unclear risk”, or “low risk”.

### Statistical analysis

A random-effects network meta-analysis was conducted by Bayesian framework. Firstly, we evaluated the global heterogeneity between treatment effects across all studies by using the I2 statistic, with values of < 25%, 25–50%, and > 50% indicating low, moderate, and high heterogeneity, respectively^11^. Secondly, analyses of residual deviance were performed to evaluate global consistency by comparing the Deviance Information Criterion (DIC) difference value between “consistency” model and “inconsistency” model^12^. In addition, node splitting was used to assess local inconsistencies when there were closed loops in the network^13^. The Surface Under the Cumulative Ranking (SUCRA) probability was the tool to estimate the ranking of each treatment^14^. Funnel plots were conducted to check publication bias of the outcomes. The Bayesian network meta-analysis was performed by the “gemtc” package in the R software^15^ through the software JAGS^16^, the pairwise meta-analysis was conducted by “meta/metagen” package in R software while version 4.1.2. STATA 14.0 and Review Manager software were used to assist graphical functions.

## Results

### Literature search and study characteristics

**Figure 1:**
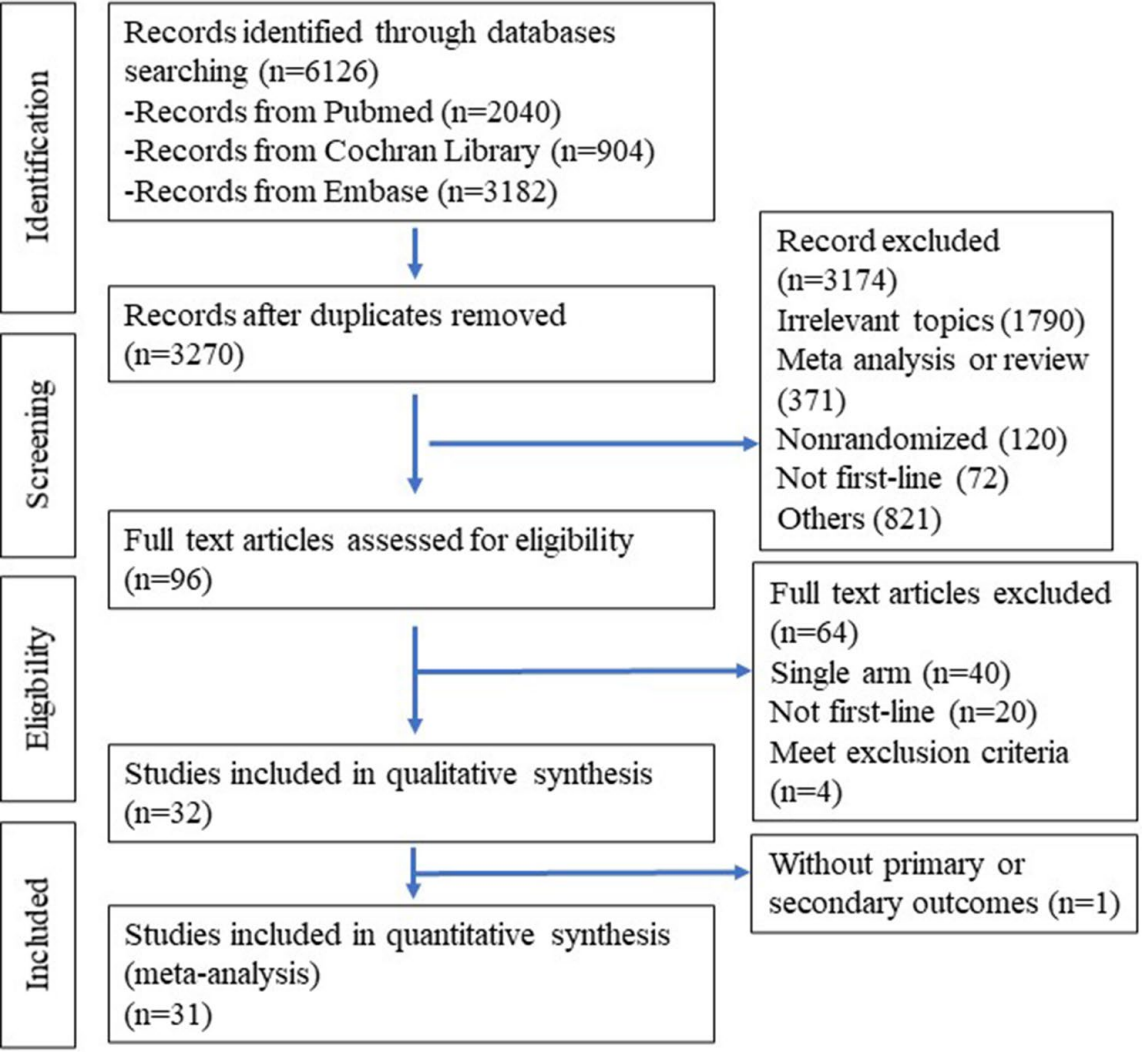
Flowchart of the study selection process.

A total of 5992 records were identified using the search strategy, and finally 96 records were selected for the full text review. Among these, 40 studies were omitted due to their single-arm design or unrandomized trials. One study was excluded because it could not be incorporated into network calculation^17^. Another study was excluded as the chemotherapy regimens did not meet the criteria for the standard first-line chemotherapy in NCCN 2021 guidelines^18^. Two other studies were not included because patients had previously received systemic chemotherapy within 6 months or the time of administration was not clearly indicated^19,20^. One study did not process in meta-analysis because of without primary and secondary outcomes reported^21^. The flow diagram of literature search is summarized in Fig. 1 and the details of reasons for exclusion are shown in Supplement Table S2. Finally, 31 RCTs were included for the network meta-analysis.

To avoid potential heterogeneity, we divided the included studies into two large subgroups. Among the 31 eligible studies, 13 studies were allocated into the “specific positivity group” analysis because these trials included patients with specific pathological positivity or PD-L1 expression (CPS ≥ 1). Meanwhile, 20 studies were included in “average group” analysis for the pathologically general population. Two studies are overlapping because the subgroup data for both the specific positivity and average groups were completely reported.

**Figure 2:**
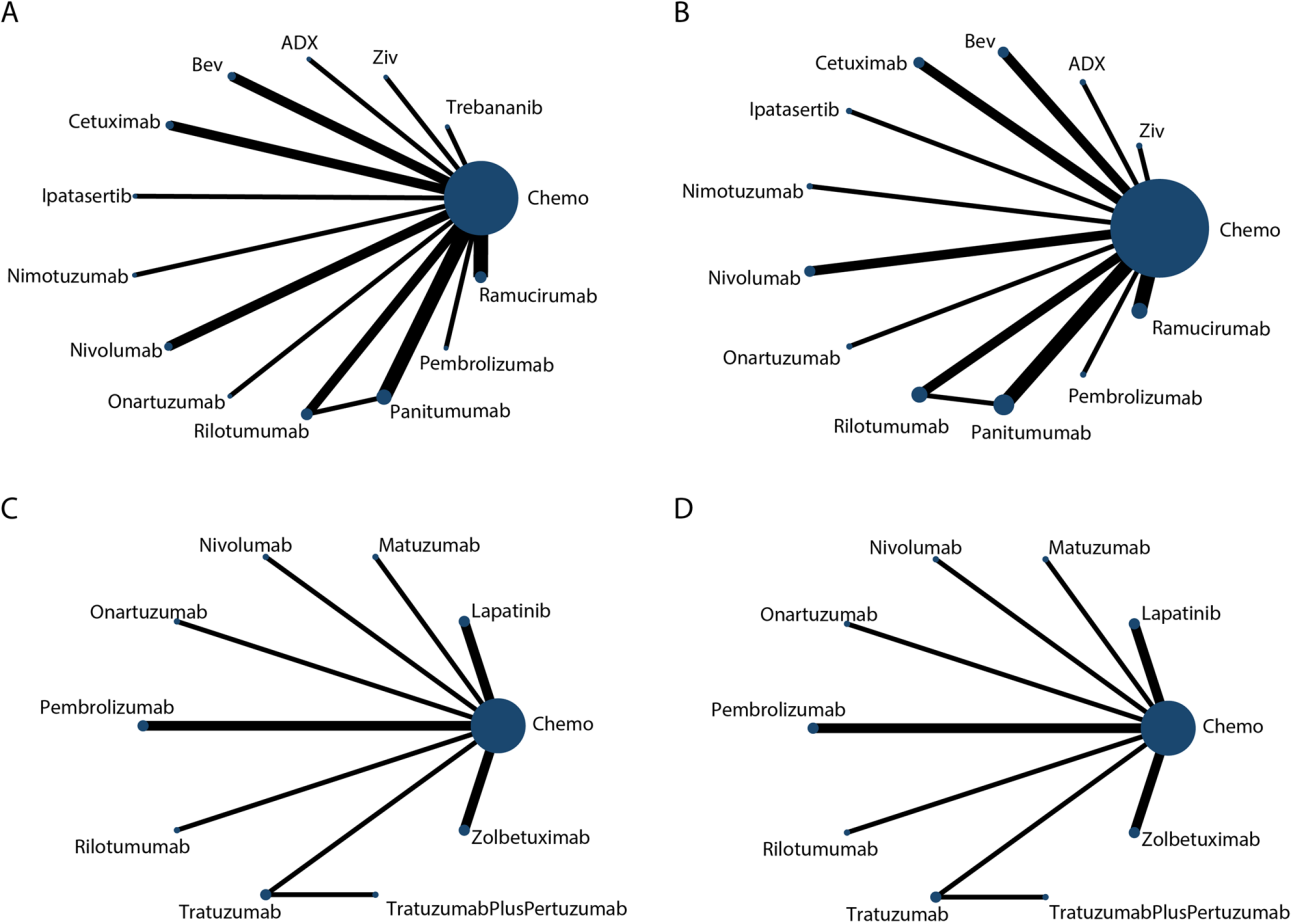
The network comparison plots of primary outcomes. (**A**) The network plots of OS in average group; (**B**) The network plots of PFS in average group; (**C**) The network plots of OS in specific positivity group; (**D**) The network plots of PFS in average group. Chemo: Chemotherapy; ADX: Andecaliximab; Ziv: Ziv-aflibercept; Bev: Bevacizumab. All regimens omitted with chemotherapy.

In average group, 20 RCTs described 13 treatment nodes. Treatment drugs included Andecaliximab (ADX), Bevacizumab (Bev), Cetuximab, Chemotherapy, Ipatasertib, Nivolumab, Nimotuzumab, Onartuzumab, Pembrolizumab, Panitumumab, Rilotumumab, Ramucirumab and Ziv-aflibercept (Ziv). For the sake of simplicity, we will use target agents or immune checkpoint inhibitors’ name instead of regimens’ full title in following. Placebo control was used in 11 trials. While 3 studies used three-drug cytotoxic regimens and others used two-drug cytotoxic regimens, all chemotherapy regimens contained fluoropyrimidine and platinum (Oxaliplatin or cisplatin). Ten trials included both Gastric cancer (GC) and Gastroesophageal junction cancer (GEJ), while 8 trials included GC, GEJ and partial esophageal cancer (EC). Among trials included EC patients, only KETNOTE-590 enrolled predominantly patients with esophageal Squamous-cell carcinoma (SCC). To avoid potential heterogeneity, we only extracted data in subgroup of adenocarcinoma (including oesophageal adenocarcinoma and gastrooesophageal junction adenocarcinoma). Three trials included AGC cases only with metastasis, while others also included locally inoperable and recurrent cases. Overall, the demographic characteristics of included trials were generally comparable. Several studies that may have introduced potential heterogeneity owing to their specific baseline features, such as three-drug cytotoxic regimens and those containing only EC and EGJ cases, were further detected in sensitivity analysis. Network plots of primary outcomes, PFS and OS, are shown in Fig. 2A,B. Characteristics of included studies are presented in Table 1.

**Table 1:** Baseline characteristics of eligible studies in average group.

Study	Regimen	Age	Region	Peritoneal involvement	Location	Advanced situation	PFS-HR	OS-HR	ORR(r/n)	AE ≥ 3 (r/n)	Note
Shan 2021 ChiCTR2000038900^21^	S-1 plus docetaxel/ cisplatin (n = 21) 2. S-1 plus docetaxel/ cisplatin plus Apatinib (n = 24)	N/A	Western/Eastern countries	N/A	GC	Locally advanced	N/A	N/A	N/A	N/A	
Shah 2021 NCT02545504 (GAMMA-1)^34^	1. Fluorouracil plus oxaliplatin plus leucovorin plus PBO (n = 214) 2. Fluorouracil plus oxaliplatin plus leucovorin plus Andecaliximab (ADX ; n = 218)	1. 63 2. 61	Europe US	N/A	GC, GEJ	Locally advanced, Metastatic	0.84 (95% CI 0.67–1.04)	0.93 (95% CI 0.74–1.18)	1. 88/214 2. 110/218	1. 108/214 2. 110/218	
Boku 2020 NCT02746796 (ATTRACTION-4)^7^	S-1/Capecitabin plus PBO (n = 362) S-1/Capecitabin plus nivolumab (n = 362)	N/A	N/A	N/A	GC, GEJ	Advanced, recurrent	0.68 (98.51%CI 0.51–0.90)	0.90 (95% CI 0.75–1.08)	1. 173/362 2. 208/362	1. 178/362 2. 210/362	HER2(−)
Kato 2020 NCT03189719 (KETNOTE-590)^34^	5-FU plus cisplatin plus PBO (n = 102) 5-FU plus cisplatin plus pembrolizumab (n = 99)	N/A	N/A	N/A	GEJ, EC (subgroup of patients with adenocarcinoma)	Locally advanced, metastatic	0.63 (95% CI 0.46–0.87)	0.74 (95% CI0.54–1.02)	1. 25/102 2. 48/99	N/A	
Mochler 2020 NCT02872116 (CheckMate649)^5^	S-1 plus oxaliplatin plus PBO (n = 792) S-1 plus oxaliplatin plus nivolumab (n = 789)	N/A	N/A	N/A	GC, GJE	Unresectable advanced, metastatic	0.77 (95% CI 0.68–0.87)	0.80 (99.3%CI 0.68–0.94)	N/A	1.77/767 2.135/782	
Yoshikawa 2019 NCT02539225 (RAINSTORM)^37^	S-1 plus oxaliplatin plus PBO (n = 93) S-1 plus oxaliplatin plus ramucirumab (n = 96)	1. 63 2. 61	Asia	1. 56 2. 63	GC, GEJ	Metastatic	1.07 (95% CI 0.86–1.33)	1.11 (95% CI0.89–1.40)	1. 47/93 2. 56/96	1. 55/93 2. 66/96	
Malka 2019 PRODIGE 17-ACCORD 20-MEGA^38^	Fluorouracil plus oxaliplatin plus leucovorin (n = 56) Fluorouracil plus oxaliplatin plus leucovorin plus panitumumab (n = 49) Fluorouracil plus oxaliplatin plus leucovorin plus rilotumumab (n = 57)	1. 64 2. 64 3. 65	Europe	N/A	GC, GEJ, EC	Locally advanced, Metastatic	0.99 (95% CI 0.77–1.27) 1.01 (95% CI 0.80–1.28)	0.99 (95% CI 0.93–1.07) 0.99 (95% CI 0.91–1.08)	1. 29/56 2. 21/49 3. 28/57	1. 33/56 2. 40/48 3. 51/57	HER-2(−)
Fuchs 2019 NCT02314117 (RAINFALL)^39^	Fluoropyrimidine plus cisplatin plus PBO (n = 319) Fluoropyrimidine plus cisplatin plus ramucirumab (n = 326)	1. 62 2. 60	Versatile	1. 111 2. 130	GC, GEJ	Metastatic	0.753 (95% CI 0.607–0.935)	0.962 (95% CI 0.801–1.156)	1. 116/326 2. 134/326	1. 160/323 2. 149/319	HER-2(−)
Cleary 2019 NCT01747551 (ZAMEGA)^40^	Fluorouracil plus oxaliplatin plus leucovorin plus PBO (n = 21) Fluorouracil plus oxaliplatin plus leucovorin plus ziv-aflibercept (n = 43)	1. 62 2. 62	Versatile	1. 7 2. 11	GC, GEJ, EC	Metastatic	1.11 (95% CI 0.64–1.91)	1.24 (95% CI0.71–2.15)	1. 16/21 2. 36/43	1. 15/21 2. 36/43	
Bang 2019 NCT01896531^41^	Fluorouracil plus oxaliplatin plus leucovorin plus PBO (n = 82) Fluorouracil plus oxaliplatin plus leucovorin plus ipatasertib (n = 71)	1. 63 2. 58	Versatile	1. 25 2. 30	GC, GEJ	Locally advanced, metastatic, recurrent	1.12 (95% CI 0.81–1.55)	1.85 (95% CI 1.23–2.79)	1. 46/82 2. 37/71	1. 61/82 2. 55/70	HER-2(−)
Yoon 2016 NCT01246960^42^	Fluorouracil plus oxaliplatin plus leucovorin plus PBO (n = 84) Fluorouracil plus oxaliplatin plus leucovorin plus ramucirumab (n = 84)	1. 60 2. 64.5	USA	N/A	GC, GEJ, EC	Locally advanced, metastatic	0.98 (95% CI 0.69–1.37)	1.08 (95% CI0.73–1.58)	1. 39/84 2. 38/84	1. 67/80 2. 74/82	
Tebbutt 2016 ATTAX3^43^	Fluoropyrimidine plus cisplatin plus docetaxel (n = 39) Fluoropyrimidine plus cisplatin plus docetaxel plus panitumumab (n = 34)	1. 59 2. 64	Australia	1. 5 2. 13	GC, GEJ, EC	Metastatic,locally recurrent	1.08 (95% CI 0.59–2.01)	1.02 (95% CI 0.51–2.05)	1. 19/39 2. 22/34	N/A	
Shah 2016 NCT01590719 (YO28252)^44^	Fluorouracil plus oxaliplatin plus leucovorin plus PBO (n = 61) Fluorouracil plus oxaliplatin plus leucovorin plus Onartuzumab (n = 62)	1. 57 2. 58.5	Asia	N/A	GC, GEJ	Inoperable, metastatic	1.08 (95% CI 0.71–1.63)	1.06 (95% CI 0.64–1.75)	1. 35/61 2. 38/62	1. 47/60 2. 53/60	HER-2(−)
Shen 2016 NCT00887822 (AVATAR)^45^	Capecitabine plus cisplatin plus PBO (n = 102) Capecitabine plus cisplatin plus Bevacizumab (n = 100)	1. 55.5 2. 54.2	Chinese	N/A	GC, GEJ	Locally advanced, metastatic, recurrent	0.89 (95% CI 0.66–1.21)	1.11 (95% CI 0.79–1.56)	1. 29/86 2. 33/81	1. 69/101 2. 60/100	
Du 2015 NCT02370849^46^	S-1 plus cisplatin (n = 31) S-1 plus cisplatin plus Nimotuzumab (n = 31)	1. 53 2. 58	Chinese	1. 5 2. 4	GC, GEJ	Locally advanced, metastatic	2.136 (95% CI 1.193–3.826)	1.776 (95% CI 0.972–3.246)	1. 18/31 2. 17/31	1. 5/31 2. 14/31	
Zhang 2014 N/A^47^	S-1 plus oxaliplatin (n = 30) S-1 plus oxaliplatin plus cetuximab (n = 27)	1. 49 2. 49	Chinese	8	GC	Unresectable or recurrence after surgery	0.67 (95% CI 0.38–1.18)	0.74 (95% CI 0.42–1.30)	1. 11/30 2. 17/27	N/A	
Iveson 2014 NCT00719550^48^	Epirubicin plus cisplatin plus capecitabine plus PBO (n = 39) Epirubicin plus cisplatin plus capecitabine plus Rilotumumab (n = 82)	1. 60 2. 60.7	Asia	N/A	GC, GEJ, EC	Unresectable locally advanced, metastatic	0.60 (95% CI 0.45–0.79)	0.70 (95% CI 0.45–1.09)	1. 8/39 2. 30/82	29/39 70/81	
Waddell 2013 NCT00824785 (REAL3)^49^	Epirubicin plus oxaliplatin plus capecitabine (n = 238) Epirubicin plus oxaliplatin plus capecitabine plus panitumumab (n = 254)	1. 62 2. 63	UK	N/A	GC, GEJ, EC	Locally advanced, metastatic	1.22 (95% CI 0.98–1.52)	1.37 (95% CI 1.07–1.76)	1. 100/238 2. 116/254	1. 166/266 2. 187/276	
Lordick 2013 EXPAND^50^	Capecitabine plus cisplatin (n = 449) Capecitabine plus cisplatin plus Cetuximab (n = 455)	1. 59 2. 60	Versatile	1. 116 2.113	GC, GEJ, EC	Locally advanced, metastatic	1.09 (95% CI 0.92–1.29)	1.00 (95% CI 0.87–1.17)	1. 131/449 2. 136/455	1. 337/436 2. 369/446	
Eatock 2013 NCT00583674^51^	Capecitabine plus cisplatin plus PBO (n = 56) Capecitabine plus cisplatin plus Trebananib (n = 115)	1. 62 2. 58.9	UK	N/A	GC, GEJ, EC	Metastatic	0.98 (95% CI 0.67–1.43)	NA	17/56 35/115	40/53 94/114	
Ohtsu 2011 NCT00548548 (AVAGAST)^52^	Capecitabine plus cisplatin plus PBO (n = 387) Capecitabine plus cisplatin plus Bevacizumab (n = 387)	1. 59 2. 58	Versatile	N/A	GC, GEJ	Locally advanced, metastatic	0.80 (95% CI 0.68–0.93)	0.87 (95% CI 0.73–1.03)	1. 111/387 2. 143/387	1. 293/381 2. 293/386	

*GC* gastric cancer, *GEJ* gastroesophageal junction cancer, *EC* esophageal cancer.

In specific positivity group, there were 11 treatment nodes among 13 RCTs. Treatment drugs included Chemotherapy, Lapatinib, Matuzumab, Nivolumab, Onartuzumab, Pembrolizumab, Rilotumumab, Trastuzumab, Trastuzumab plus Pertuzumab, Trastuzumab plus Pembrolizumab and Zolbetuximab. Six trials used placebo control while others used an open-label design. Five trials chose a three-drug cytotoxic regimen, Epirubicin plus fluoropyrimidine plus platinum, while others used a two-drug regimen containing fluoropyrimidine plus platinum. Three trials included partial lower EC cases, while 1 trial included EC and GEJ without any GC patients, we only analysis data in subgroup of esophageal adenocarcinoma. Three trials included AGC cases only with metastasis, while others also included locally inoperable and recurrent cases. To confirm the comparable baseline, studies with potential heterogeneity were checked for their influence by sensitivity analysis. Network plots of primary outcomes, PFS and OS, are presented in Fig. 2C,D. Baseline characteristics of included studies are summarized in Table 2.

**Table 2:** Baseline characteristics of eligible studies in specific positivity group.

Study	Regimen	Age	Region	Peritoneal involvement	Location	Advanced situation	PFS-HR	OS-HR	ORR(r/n)	AE ≥ 3 (r/n)	Note
Janjigan 2021 NCT03615326 (KEYNOTE-811)^24^	1. Pembrolizumab plus trastuzumab plus Cisplatin/Oxaliplatin plus fluorouraciln(n = 133) 2. Trastuzumab plus Cisplatin/Oxaliplatin plus fluorouraciln(n = 131)	N/A	N/A	NA/A	GC, GJE	Unresectable, metastatic	N/A	N/A	1. 99/133 2. 68/131	1. 124/217 2. 124/216	HER-2(+)
Sahin 2021 NCT01630083 (FAST)^30^	1. Epirubicin plus oxaliplatin plus capecitabine (n = 84) 2. Epirubicin plus oxaliplatin plus capecitabine plus zolbetuximab (n = 77)	1. 57 2. 59	N/A	1. 23 2. 20	GC, GEJ, EC	Locally advanced, inoperable, recurrent, metastatic	0.44 (95% CI 0.29–0.67)	0.55 (95% CI 0.39–0.77)	1. 21/84 2. 30/77	1. 54/84 2. 54/77	CLDN18.2 expression ≥ 40%
Shitara 2020 NCT02494583 (KEYNOTE-062)^33^	1. Cisplatin plus fluorouraciln plus PBO (n = 250) 2. Cisplatin plus fluorouraciln plus pembrolizumab (n = 257)	1. 62.5 2. 62	Versatile	N/A	GC, GEJ	Locally advanced/unresectable, metastatic	0.84 (95% CI 0.70–1.02)	0.85 (95% CI 0.70–1.03)	1. 93/250 2. 125/257	1. 169/250 2. 183/257	CPS ≥ 1
Kato 2020 NCT03189719 (KEYNOTE-590)^34^	1. 5-FU plus cisplatin plus PBO (n = 54) 2. 5-FU plus cisplatin plus Pembrolizumab (n = 43)	N/A	N/A	N/A	GEJ, EC	Locally advanced, metastatic	0.49 (95% CI, 0.30–0.81)	0.83 (95% CI 0.52–1.34)	N/A	N/A	CPS ≥ 10
Mochler 2020 NCT02872116 (CheckMate649)^5^	1. S-1 plus oxaliplatin plus PBO (n = 465) 2. S-1 plus oxaliplatin plus nivolumab (n = 468)	N/A	N/A	N/A	GC, GJE	Unresectable advanced, metastatic	0.68 (95% CI, 0.56–0.81)	0.71 (95% CI, 0.59–0.86)	N/A	1. 203/465 2. 277/468	CPS ≥ 5
Tabernero 2018 NCT01774786 (JACOB)^25^	1. Cisplatin plus fluorouraciln plus trastuzumab (n = 392) 2. Cisplatin plus fluorouraciln plus trastuzumab plus pertuzumab (n = 388)	1. 61 2. 62	Versatile	N/A	GC, GEJ	Metastatic	0.73 (95% CI 0.62–0.86)	0.84 (95% CI 0.71–1.00)	1. 189/392 2. 220/388	1. 282/388 2. 307/388	HER-2(+) IHC 3+/IHC 2+
Mochler 2018 NCT01123473^27^	1. Epirubicin plus cisplatin plus 5-fluorouracil/capecitabine plus PBO (n = 14) 2. Epirubicin plus cisplatin plus 5-fluorouracil/capecitabine plus Laptinib (n = 14)	1. 58 2. 66	Europe	N/A	GC, GEJ	Unresectable, metastatic	0.86 (95% CI0.37–1.99)	0.90 (95% CI0.35–2.27)	1. 3/14 2. 6/14	1. 7/14 2. 9/14	HER2(+)/EGFR(+)
Shah 2017 NCT01662869^31^	1. Fluorouracil plus oxaliplatin plus leucovorin plus PBO (283) 2. Fluorouracil plus oxaliplatin plus leucovorin plus Onartuzumab (n = 279)	1. < 65: 189; > 65: 94 2. < 65: 183; > 65: 96	Versatile	No	GC, GEJ	Metastatic	0.90 (95% CI 0.71–1.16)	0.82 (95% CI0.59–1.15)	1. 84/207 2. 100/217	1. 187/279 2. 192/280	MET(2+ 3+)
Catenacci 2017 NCT01697072 (RILOMET-1)^32^	1. Epirubicin plus cisplatin plus capecitabine plus PBO (n = 305) 2. Epirubicin plus cisplatin plus capecitabine plus Rilotumumab (n = 304)	1. 59 2. 61	Versatile	N/A	GC, GEJ	Locally advanced, metastatic, recurrent	1.26 (95% CI 1.04–1.51)	1.34 (95% CI 1.10–1.63)	1. 119/267 2. 78/262	1. 149/299 2. 142/298	MET ≥ (1+)
Schuler 2016 NCT01246960^29^	1. Epirubicin plus oxaliplatin plus capecitabine (n = 161) 2. Epirubicin plus oxaliplatin plus capecitabine plus zolbetuximab (n = 161)	Median: 58	Europe	N/A	GC, GEJ	Locally advanced, metastatic, recurrent	0.47 (95% CI 0.31–0.70)	0.51 (95% CI0.36–0.73)	1. 45/161 2. 62/161	N/A	CLDN18.2
Hecht 2016 NCT00680901 (TRIO013/LOGiC)^28^	1. Capecitabine Plus Oxaliplatin (n = 267) 2. Capecitabine Plus Oxaliplatin plus lapatinib (n = 270)	1. 59 2. 61	Versatile	N/A	GC, GEJ, EC	Unresectable	0.82 (95% CI 0.68–1.0)	0.91 (95% CI 0.73–1.12)	1. 93/238 2. 131/249	1. 52/267 2. 72/270	HER2(+)/EGFR(+)
Rao 2010 NCT0021564436^53^	1. Epirubicin plus cisplatin plus capecitabine (n = 36) 2. Epirubicin plus cisplatin plus capecitabine plus Matuzumab (n = 35)	1. 64 2. 69	Europe	1. 25 2. 29	GC, GEJ, EC	Metastatic	1.13 (95% CI 0.63–2.01)	1.02 (95% CI 0.61–1.70)	1. 21/36 2. 11/35	1. 25/36 2. 27/35	EGFR(+)
Bang 2010 NCT01041404 (ToGA)^23^	1. Capecitabine/5-FU plus cisplatin (n = 290) 2. Capecitabine/5-FU plus cisplatin plus Trastuzumab (n = 294)	1. 58.5 2. 59.4	Versatile	N/A	GC, GEJ	Locally advanced, metastatic, recurrent	0.71 (95% CI 0.59–0.85)	0.74 (95% CI 0.60–0.91)	1. 100/294 2. 139/294	1. 198/290 2. 201/294	HER2(+)

*GC* gastric cancer, *GEJ* gastroesophageal junction cancer, *EC* esophageal cancer.

### Risk of bias assessment

**Figure 3:**
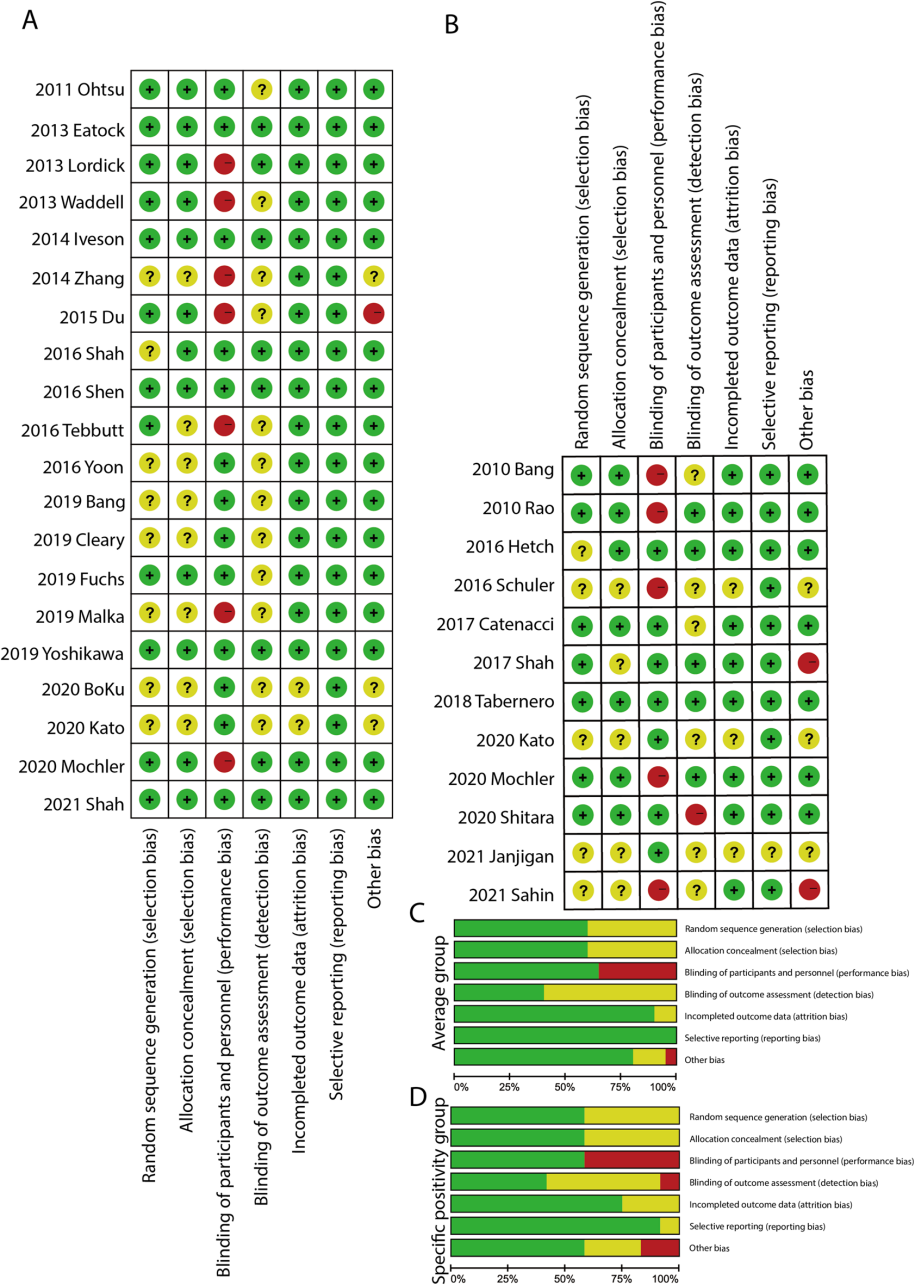
Risk of bias assessment. (**A**) Risk of bias assessment plots in average group; (**B**) Risk of bias plots assessment in specific positivity group; (**C**) Risk of bias proportion in average group; (**D**) Risk of bias proportion in specific positivity group.

Generally, the risk of bias was low in the 31 included studies. The primary source of high-risk bias was in the domain of blinding of participants and personnel due to the open-label design, which resulted in 39.39% of the studies scoring as high-risk of bias. Meanwhile, 9.09% of the trials had a high risk of bias mostly due to an early termination of patient recruitment. The summary of bias is shown in Fig. 3A–D, and the detailed assessment of each study is shown in supplement Tables S3 and S4.

### Heterogeneity, consistency and publication bias

Statistical heterogeneity was low across the studies for primary and secondary outcomes in both average group and specific positivity group (all *I*^2^ < 25%, ranging from 0.0005 to 6%) by fitting a random-effects model. The differences in values of DIC in both “consistency” and “inconsistency” models were used to evaluate the global consistency. In all outcomes the differences in DIC values were low, ranging from 0.007 to 0.15, which indicates a good level of global consistency. The summary of *I*^2^ and DIC value showed in supplemental Table S5. Local consistency analysis was only conducted in the average group because specific positivity group had no closed loops for comparison. The p-values of indirect and direct comparisons between Rilotumumab and Panitumumab were 0.14, 0.09, 0.65 and 0.91 for OS, PFS, ORR and AE ≥ 3, respectively, which indicates no significant local inconsistency. There was no publication bias among the included studies both in the average group and specific positivity group, which can be seen from the symmetrical distribution of effect sizes in the funnel plots (Supplementary Figs. S3 and S4).

### Primary outcome: progression-free survival (PFS)

**Figure 4:**
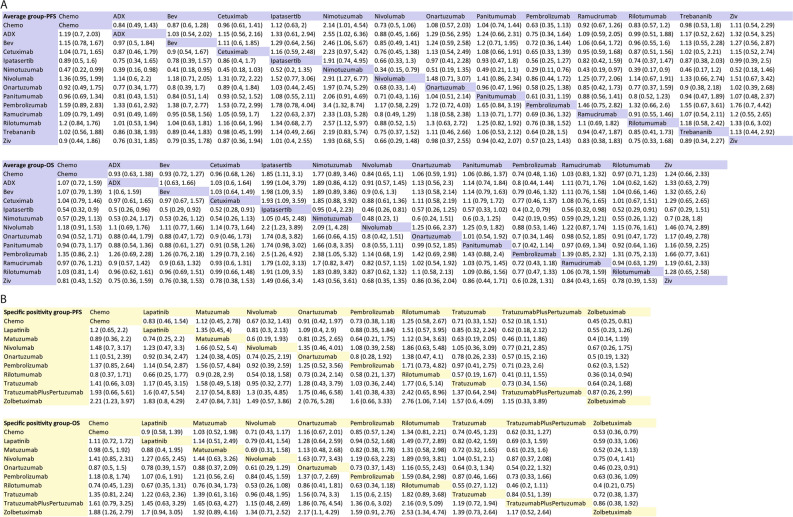
Network league table for secondary outcomes. (**A**) League table for PFS and OS in average group; (**B**) League table for PFS and OS in specific positivity group.

**Figure 5:**
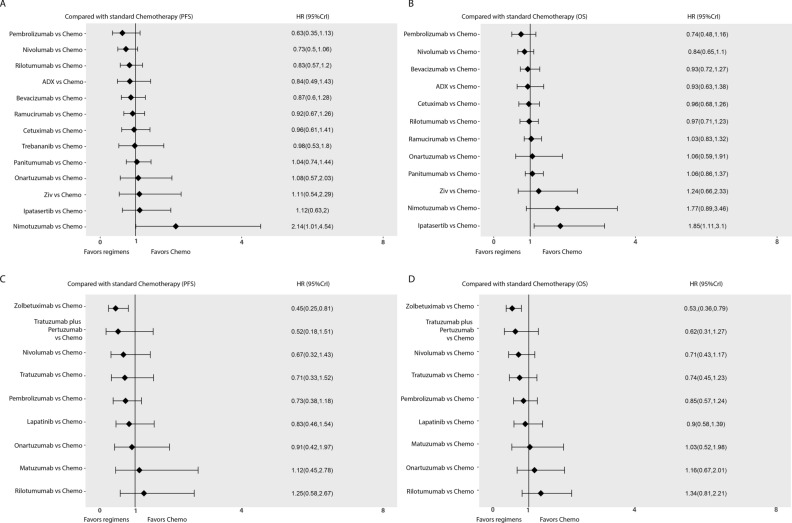
Forest plots of primary outcomes compared with Standard Chemotherapy. (**A**) Forest plots of PFS compared with chemotherapy in average group; (**B**) Forest plots of OS compared with chemotherapy in average group; (**C**, Forest plots of PFS compared with chemotherapy in specific positivity group; (**D**) Forest plots of OS compared with chemotherapy in specific positivity group.

**Figure 6:**
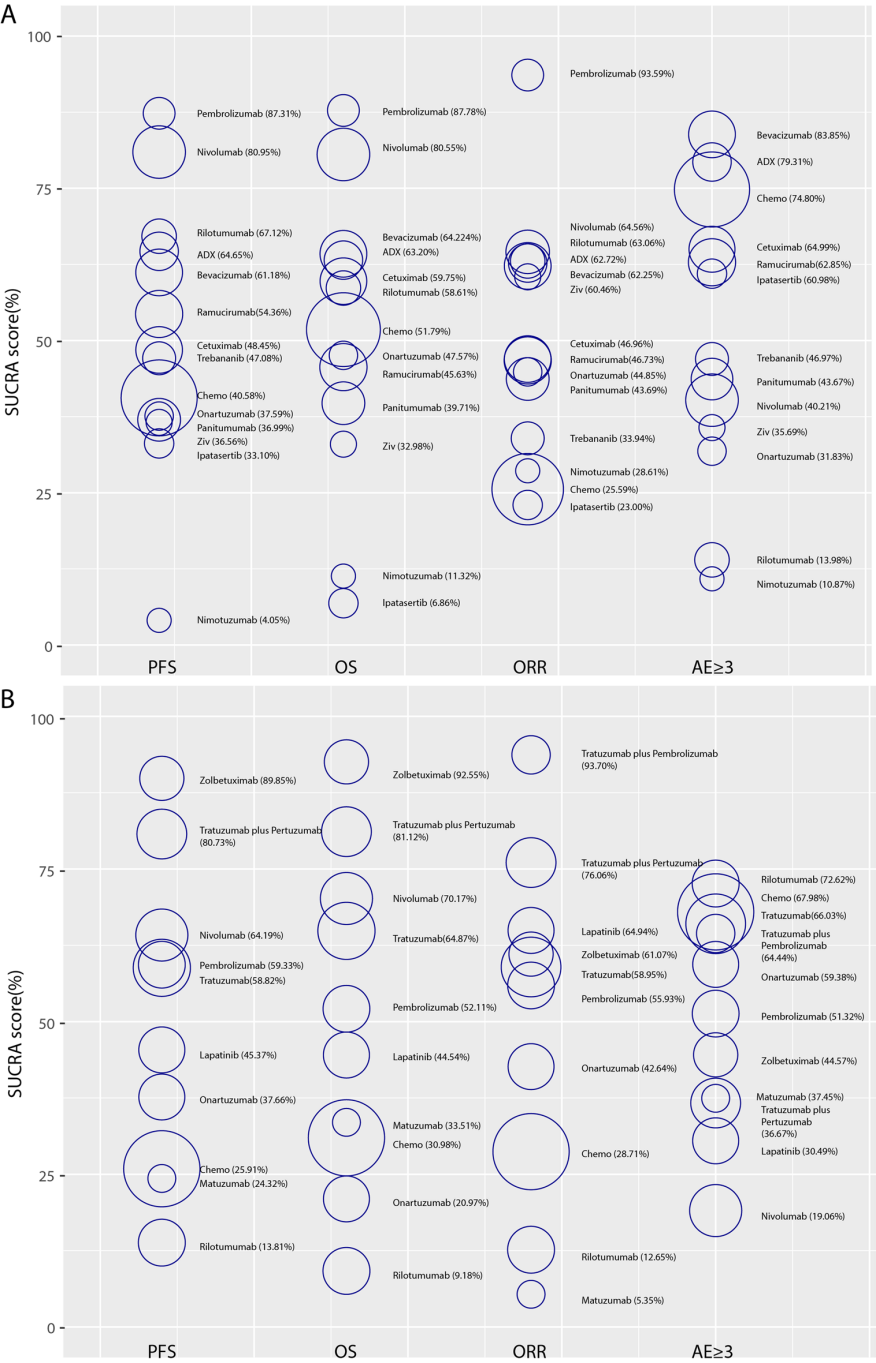
SUCRA score of each regimen in all outcomes. (**A**) SUCRA score in average group; (**B**) SUCRA score in specific positivity group. The size of each circle is weighted by the square root of the patient number. All regimens are combined with chemotherapy, ADX: Andecaliximab; Ziv: Ziv-aflibercept; Chemo: Chemotherapy.

With respect to PFS in average group for network meta-analysis, there are 20 trails containing 13 separated nodes. No regimen showed an obviously improvement than standard chemotherapy, although nivolumab was very close to statistical significance (HR 0.73, 95% CI 0.5–1.06). Pembrolizumab, Rilotumumab, ADX and bevacizumab, also showed improvement compared to standard chemotherapy (HR 0.63, 95% CI 0.35–1.13; HR 0.83, 95% CI 0.57–1.2; HR 0.84, 95% CI 0.49–1.43; HR 0.87, 95% CI 0.6–1.28, respectively). All other regimens were comparable to standard chemotherapy except nimotuzumab, which had inferiority effect than standard chemotherapy alone (Fig. 5A). League table summarizing the direct and indirect comparisons between the regimens is shown in Fig. 4A. Furthermore, from SUCRA score of PFS, Pembrolizumab (87.31%) was ranked first in improving PFS, followed by nivolumab (80.59%) and rilotumumab (67.12%), while nimotuzumab was ranked last (4.05%) (Fig. 6A).

In specific positivity group, the network plot analysis was the same as OS results. Zolbetuximab showed a significant improvement in PFS (HR 0.45, 95% CI 0.25–0.81). Trastuzumab, trastuzumab plus pertuzumab, lapatinib, pembrolizumab and nivolumab, showed certain extend of advantage in PFS than standard chemotherapy. Except rilotumumab, other regimens were comparable to standard chemotherapy (Fig. 5C). League table summarizing all comparisons between the regimens is shown in Fig. 4B. Furthermore, in the rank of SUCRA score, zolbetuximab, trastuzumab plus pertuzumab and nivolumab occupied the top three ranks (89.85%, 80.73% and 64.19% respectively), while rilotumumab, with its score at 13.81%, was ranked last (Fig. 6B).

### Primary outcome: overall survival (OS)

In the network meta-analysis of OS, 19 trials containing 13 separated nodes in the average group reported the primary outcomes of OS. Unfortunately, no regimen had a statistically significant difference in prolonging the OS in comparison to chemotherapy. Two immunotherapy drugs, pembrolizumab and nivolumab, were close to survival advantage significance (HR 0.74, 95% CI 0.48–1.16; HR 0.84, 95% CI 0.65–1.11, respectively), while others were comparable to standard chemotherapy except two poor effect regimens, nimotuzumab and ipatasertib. (Fig. 5B). Results of different treatments in both direct and indirect comparisons are shown in a league table (Fig. 4A). In addition, we ranked the comparative effects of all regimens based on their SUCRA values: pembrolizumab (87.78%) was the most likely to improve OS, followed by nivolumab (80.55%) and bevacizumab (64.22%), while ipatasertib was ranked last (6.86%) (Fig. 6A).

In specific positivity group, 12 trials reported the endpoint of OS, including 10 independent nodes. Zolbetuximab was the only regimen with a significant difference from standard chemotherapy (HR 0.53, 95% CI 0.36–0.79). Trastuzumab, trastuzumab plus pertuzumab, pembrolizumab and nivolumab, showed a certain extend improvement for OS compared to standard chemotherapy (Fig. 5D). Onartuzumab, matuzumab, and lapatinib, were comparable to standard chemotherapy, while rliotumumab had a more negative effect on OS (Fig. 5D). League table summarizing all comparisons between the regimens is shown in Fig. 4B. Ultimately, taking into account the comparative effects of all regimens regarding OS, zolbetuximab, trastuzumab plus pertuzumab and nivolumab occupied top three by the SUCRA score (92.55%, 81.12% and 70.17% respectively), while rilotumumab came bottom (9.18%) (Fig. 6B).

### Secondary outcomes: objective response rate (ORR) and adverse events (AEs) ≥ 3

A total of 19 and 11 studies in average group and specific positivity group were eligible and merged for the analysis of ORR. From the result of interval comparisons, only pembrolizumab in average group revealed a significant advantage compared to standard chemotherapy (RR 2.93, 95% CI 1.25–6.89), although pembrolizumab plus trastuzumab was very close to statistical significance (RR 4.75, 95% CI 0.99–22.69) (Supplement Fig. S5A). Pembrolizumab, nivolumab and rilotumumab were ranked at the top three by SUCRA scores in average group (93.59%, 64.56% and 63.06%, respectively) (Fig. 6A), meanwhile pembrolizumab plus trastuzumab (93.70%) was the best, followed by trastuzumab plus pertuzumab (76.06%) and lapatinib (64.94%) in specific positivity group (Fig. 6B). In the analysis of AE ≥ 3 outcomes, 18 and 10 studies in average group and specific positivity group respectively were included. The safest regimens were revealed to be bevacizumab, ADX, and chemotherapy in average group by SUCRA score (83.85%, 79.31% and 74.80% respectively) (Fig. 6A) while nimotuzumab was ranked at the bottom (10.87%). In specific positivity group, the highest three were rilotumumab, chemotherapy and trastuzumab (72.62%, 67.98% and 66.03% respectively), while the lowest-ranked regimen was nivolumab (19.06%) (Fig. 6B).

### Pairwise meta-analysis of specific positivity group

**Figure 7:**
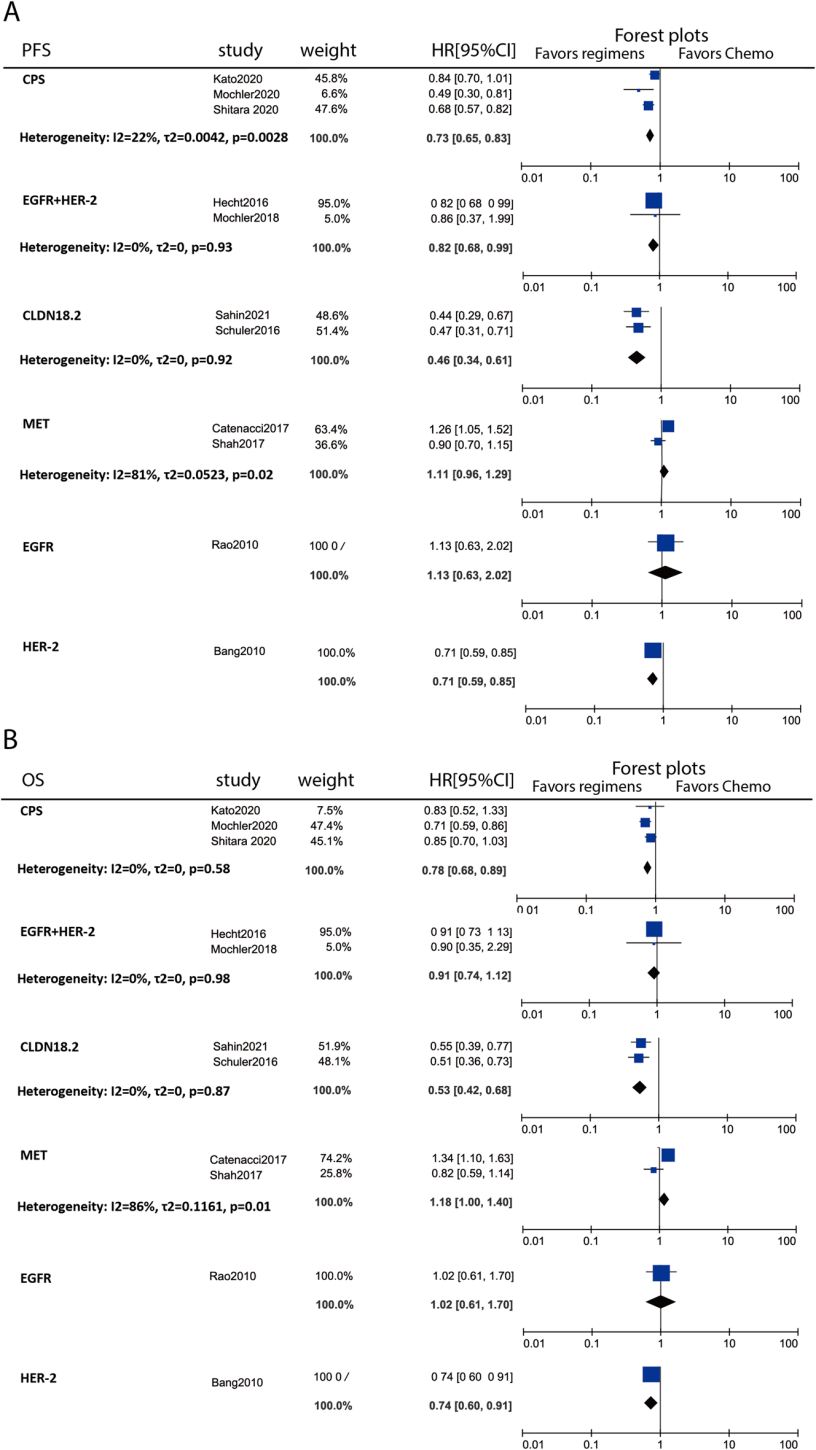
Pairwise meta-analysis forest plots for specific positivity group. (**A**) Forest plots of PFS; (**B**) Forest plots of OS. All regimens are compared with chemotherapy.

Based on the overall indirect comparisons of all regimens in specific positivity group, we next explore the primary outcomes in each pathological positivity conducted by pairwise meta-analysis in random model. 12 trials including three PD-L1 CPS positive studies, two EGFR plus HER-2 positive studies, two CLDN18.2 positive studies, two MET-1 positive studies, one HER-2 positive study (Tabernero 2018 excluded here due to no chemotherapy arm), and one EGFR positive study (Fig. 7A,B). From the result, we found that CPS positive patients’ survival will be benefit from immune check point inhibitor regimens, both OS (HR 0.78, 95% CI 0.68–0.89) and PFS (HR 0.73, 95% CI 0.65–0.83) showed significantly increase. As well as IMAB362, which markedly enhanced survival benefits among CLDN18.2 positive patients (OS: HR 0.53, 95% CI 0.42–0.68; PFS: HR 0.46, 95% CI 0.34–0.61). Among both HER-2 and EGFR positive patients, lapatinib, dual tyrosine kinase inhibitor which interrupts the HER-2 and EGFR pathways, failed to produce survival benefits (OS: HR 0.91, 95% CI 0.74–1.12; PFS: HR 0.83, 95% CI 0.68–1.00). Identically, matuzumab, reported by Rao 2010, showed no significantly advantage in survival improvement among EGFR positive patients. While According to Bang 2010 trial, trastuzumab could significantly enhance its survival benefits among HER-2 positive patients. In addition, MET positive patients were hardly to be benefited from MET target agents, as summary of rilotumumab and onartuzumab, OS (HR 1.18, 95% CI 1.00–1.40) and PFS (HR 1.11, 95% CI 0.96–1.29) even showed it not only failed to increase but also significantly decreased the survival time.

## Discussion

In this study, we performed a Bayesian network meta-analysis to analyze the efficacy and tolerability in untreated AGC patients who received target agents or immune checkpoint inhibitors along with chemotherapy as first-line treatments. We divided the included studies into average and specific positivity groups, which was based on whether the study population had specific pathological positivity or a certain PD-L1 CPS. As a result, immune checkpoint inhibitors (pembrolizumab and nivolumab) offered survival benefits among average patients, while had a higher AE rate than standard chemotherapy. In specific positivity group, zolbetuximab showed obviously improvement in OS and PFS among CLDN18.2 positive patients, meanwhile, when patients had positive CPS expression, they will get benefits from immune checkpoint inhibitors (pembrolizumab and nivolumab) therapy, with moderate AE rate.

In general, among the average group, none of the target agent treatments showed a significant superiority compared to standard chemotherapy. Although there were 6 and 8 regimens that ranked higher than chemotherapy in OS and PFS respectively, the HRs of OS and PFS were still no significance compared to chemotherapy. In 2014, American Society of Clinical Oncology (ASCO) expert meeting stated that a risk reduction of HR 0.80 might be clinically relevant with metastatic disease^22^. Therefore, in consideration of survival efficacy and safety profile, it is appropriate to conclude that among the existing target agents, there are no regimens superior to first-line standard chemotherapy in the population of patients who are not selected by specific pathological positivity. However, immunotherapy combined with chemotherapy showed potential benefits, especially nivolumab, which HR and 95% CI of PFS were very close to statistical significance in our meta-analysis, indicating that immune checkpoint therapy on PD-1 receptor may have a positive effect on PFS, which may bring about a promising direction for general patients.

In specific positivity group, the diversity of targets positivity may contribute to the source of heterogeneity therefore infect the transitivity assumption. To address this, we evaluated statistical heterogeneity of the network meta-analysis by *I*^2^ statistic. Neither primary or secondary outcomes showed potential heterogeneity (all *I*^2^ ≤ 5%), indicated that transitivity assumption is sound among specific group. Since Bang et al*.*^23^ reported the largescale phase III RCT ToGA, HER-2 gradually evolved as the most widely investigated target against AGC. The addition of trastuzumab to standard chemotherapy has been confirmed as the first-line therapy among AGC patients with HER-2 overexpression. Meanwhile, KEYNOTE-811^24^ had reported the combination of pembrolizumab, trastuzumab and chemotherapy, which provided a substantial, statistically significant improvement in ORR compared with placebo, trastuzumab, and chemotherapy for HER2-positive advanced gastric cancer patients. In our meta-analysis, it was the first at the rank of ORR in specific positivity group. Although initial data did not report OS and PFS, we will continuously concern the following results. Moreover, surrounding HER-2 target, a series of RCT have been reported over decade, but outcomes were diversity. Pertuzumab, a monoclonal HER2-targeted antibody which binds to different epitope in HER-2 receptor than trastuzumab^25^, has been shown to signifcantly improve survival outcomes when added to trastuzumab plus chemotherapy in patients with HER2-positive metastatic breast cancer^26^. However, the dual HER-2 targeted therapy failed to generate OS benefit than trastuzumab based regimen, despite the difference of OS coming close to crossing the boundary value (OS HR: 0.84 (95% CI, 0.71–1.00)). In addition, lapatinib, dual tyrosine kinase inhibitor which interrupts the HER-2 and EGFR pathways, failed to produce survival benefits^27,28^. In our study, neither network or pairwise meta-analysis showed the higher-rank or survival significance than other regimens. Above results indicated the inherent differences in tumor biology between gastric cancer and breast cancer, the potential role for HER-2 in gastric cancer progression should be further explore.

In the pooled result of Schuler et al.^29^ and Sahin et al.^30^, the addition of zolbetuximab (IMAB362) significantly elongated OS and PFS among patients with CLDN18.2 positivity compared with triplet chemotherapy alone, which indicates that zolbetuximab may be a promising medication for AGC patients. Unfortunately, adding rilotumumab or onartuzumab failed to generate survival benefits among MET-1 positive patients from both network and pairwise meta-analysis^31,32^. This suggests that standard first-line chemotherapy may still serve as the preferred first-line regimen in MET-1 positive AGC patients.

In the selected population with PD-L1 expression, immune checkpoint inhibitors of PD-1 also revealed their survival benefits. The addition of nivolumab to standard first-line chemotherapy obviously prolonged OS and PFS among patients who had CPS of 5 or higher. This regimen was also recommended as first-line therapy in HER-2-negative patients in NCCN 2021 guideline^4^. Our network meta-analysis is consistent with this conclusion in that among patients without HER-2 and CLDN18.2 positivity, nivolumab was the preferred option, which is shown by the SUCRA ranks. Although nivolumab has higher risk of AE ≥ 3 than chemotherapy, there was no statistical significance and fatal AEs are reported to be very rare. Another PD-1 checkpoint inhibitor, pembrolizumab, with chemotherapy, also showed some superiority in OS and PFS over standard chemotherapy. In RCT KEYNOTE-062^33^, whose patients had GC and GEJ, with CPS of 1 or greater, the difference in OS was quite close to the statistical boundary. Meanwhile, KEYNOTE-590^34^, which included GEJ and EC patients with CPS of 10 or higher, showed a significant improvement in survival benefits. After pooling these two RCTs together, pembrolizumab plus chemotherapy was ranked second when patients had no HER-2 and CLDN18.2 overexpression. Targeted agents or immune checkpoint inhibitors as monotherapy was common in second or third line advanced gastric cancer treatment. Monotherapy of ramucirumab was recommended as second-line therapy and pembrolizumab as third-line therapy in NCCN 2021 guideline^4^. Since chemotherapy is standard treatment in first-line therapy of advanced gastric cancer, clinical trials of targeted agents or immune checkpoint inhibitors as monotherapy were rare. However, there are still some phase II/III clinical trials which can give us new insight^35,36^. Pembrolizumab monotherapy was one arm in KEYNOTE-062^33^ trial, investigated as first-line treatment. Trial reported that pembrolizumab was noninferior but not superior to chemotherapy for OS in patients with CPS of 1 or greater, while prolonged OS in patients CPS ≥ 10. Meanwhile, pembrolizumab monotherapy showed less AEs grade 3–5 than chemotherapy (17% vs 69%), indicated comparable efficiency but higher safety. Will monotherapy of immune checkpoint inhibitors become first-line treatment for advanced gastric cancer? Except for waiting more high-quality RCTs, economic cost will be a very important concern.

## Conclusions

In conclusion, among average patients who were not selected by pathological positivity or PD-L1 expression, immune checkpoint inhibitor of PD-1 plus chemotherapy will be the promising regimen. Patients who have the overexpression of CLDN18.2, zolbetuximab combined with chemotherapy has higher survival benefits. Furthermore, for patients who have PD-L1 expression with no HER-2 or CLDN18.2 positivity, additional immune checkpoint inhibitor of PD-1 will be a good considered option.

## Supplementary Information

Supplementary Information.

## Data Availability

All data generated or analyzed during this study are included in this article and its additional information files. The datasets and codes are also available from the corresponding author on reasonable request.

## References

[1] Sung H (2021). Global cancer statistics 2020: GLOBOCAN estimates of incidence and mortality worldwide for 36 cancers in 185 countries. CA Cancer J. Clin..

[2] Ferlay J (2021). Cancer statistics for the year 2020: An overview. Int. J. Cancer..

[3] Van Cutsem E, Sagaert X, Topal B, Haustermans K, Prenen H (2016). Gastric cancer. Lancet.

[4] Ajani JA (2022). Gastric cancer, version 2.2022, NCCN clinical practice guidelines in oncology. J. Natl. Compr. Cancer Netw..

[5] Janjigian YY (2021). First-line nivolumab plus chemotherapy versus chemotherapy alone for advanced gastric, gastro-oesophageal junction, and oesophageal adenocarcinoma (CheckMate 649): A randomised, open-label, phase 3 trial. Lancet.

[6] Cheng J (2019). First-line systemic therapy for advanced gastric cancer: A systematic review and network meta-analysis. Ther. Adv. Med. Oncol..

[7] Xie S, Zhang H, Wang X, Ge Q, Hu J (2017). The relative efficacy and safety of targeted agents used in combination with chemotherapy in treating patients with untreated advanced gastric cancer: A network meta-analysis. Oncotarget.

[8] Tierney JF, Stewart LA, Ghersi D, Burdett S, Sydes MR (2007). Practical methods for incorporating summary time-to-event data into meta-analysis. Trials.

[9] Woods BS, Hawkins N, Scott DA (2010). Network meta-analysis on the log-hazard scale, combining count and hazard ratio statistics accounting for multi-arm trials: A tutorial. BMC Med. Res. Methodol..

[10] Cumpston, M. S., McKenzie, J. E., Welch, V. A. & Brennan, S. E. Strengthening systematic reviews in public health: Guidance in the Cochrane Handbook for Systematic Reviews of Interventions, 2nd edition. *J Public Health (Oxf)*. 10.1093/pubmed/fdac036 (2022). 10.1093/pubmed/fdac036 PMC9715291 35352103

[11] Higgins JP, Thompson SG, Deeks JJ, Altman DG (2003). Measuring inconsistency in meta-analyses. BMJ.

[12] White IR, Barrett JK, Jackson D, Higgins JP (2012). Consistency and inconsistency in network meta-analysis: Model estimation using multivariate meta-regression. Res. Synth. Methods.

[13] van Valkenhoef G, Dias S, Ades AE, Welton NJ (2016). Automated generation of node-splitting models for assessment of inconsistency in network meta-analysis. Res. Synth. Methods.

[14] Salanti G, Ades AE, Ioannidis JP (2011). Graphical methods and numerical summaries for presenting results from multiple-treatment meta-analysis: An overview and tutorial. J. Clin. Epidemiol..

[15] Team., R. C. *R: A Language and Environment for statistical computing. R Foundation for Statistical Computing*. https://www.R-project.org/ (2021).

[16] Plummer, M. *Just another Gibbs sampler (JAGS) version 4.3.0*. https://mcmc-jags.sourceforge.io/ (2017).

[17] Boku N (2019). Safety and efficacy of nivolumab in combination with S-1/capecitabine plus oxaliplatin in patients with previously untreated, unresectable, advanced, or recurrent gastric/gastroesophageal junction cancer: Interim results of a randomized, phase II trial (ATTRACTION-4). Ann. Oncol..

[18] Li C, Tang T, Wang W (2020). Combination use of tegafur and apatinib as first-line therapy in treatment of advanced gastric cancer: A single-blinded randomized study. Gastroenterol. Res. Pract..

[19] Richards D (2013). Results of docetaxel plus oxaliplatin (DOCOX) ± cetuximab in patients with metastatic gastric and/or gastroesophageal junction adenocarcinoma: Results of a randomised Phase 2 study. Eur. J. Cancer (Oxford, England : 1990).

[20] Koizumi W (2013). Randomised phase II study of S-1/cisplatin plus TSU-68 vs S-1/cisplatin in patients with advanced gastric cancer. Br. J. Cancer.

[21] Shan Z (2021). A prospective, randomized, controlled, multicenter study of apatinib combined with S-1 plus docetaxel as adjuvant therapy for locally advanced gastric cancer. J. Clin. Oncol..

[22] Ellis, L. M. *et al.* American Society of Clinical oncology perspective: Raising the bar for clinical trials by defining clinically meaningful outcomes. *J. Clin. Oncol.* **32**, 1277-+. 10.1200/Jco.2013.53.8009 (2014). 10.1200/JCO.2013.53.8009 24638016

[23] Bang YJ (2010). Trastuzumab in combination with chemotherapy versus chemotherapy alone for treatment of HER2-positive advanced gastric or gastro-oesophageal junction cancer (ToGA): A phase 3, open-label, randomised controlled trial. Lancet.

[24] Janjigian Y (2021). Initial data from the phase 3 KEYNOTE-811 study of trastuzumab and chemotherapy with or without pembrolizumab for HER2-positive metastatic gastric or gastroesophageal junction (G/GEJ) cancer. Ann. Oncol..

[25] Tabernero J (2018). Pertuzumab plus trastuzumab and chemotherapy for HER2-positive metastatic gastric or gastro-oesophageal junction cancer (JACOB): Final analysis of a double-blind, randomised, placebo-controlled phase 3 study. Lancet Oncol..

[26] Swain SM (2013). Pertuzumab, trastuzumab, and docetaxel for HER2-positive metastatic breast cancer (CLEOPATRA study): Overall survival results from a randomised, double-blind, placebo-controlled, phase 3 study. Lancet Oncol..

[27] Moehler M (2018). Lapatinib with ECF/X in the first-line treatment of metastatic gastric cancer according to HER2neu and EGFR status: A randomized placebo-controlled phase II study (EORTC 40071). Cancer Chemother. Pharmacol..

[28] Hecht JR (2016). Lapatinib in combination with capecitabine plus oxaliplatin in human epidermal growth factor receptor 2-positive advanced or metastatic gastric, esophageal, or gastroesophageal adenocarcinoma: TRIO-013/LOGiC–A randomized phase III trial. J. Clin. Oncol..

[29] Schuler M (2016). Final results of the FAST study, an international, multicenter, randomized, phase II trial of epirubicin, oxaliplatin, and capecitabine (EOX) with or without the anti-CLDN18.2 antibody IMAB362 as first-line therapy in patients with advanced CLDN18.2+ gastric and gastroesophageal junction (GEJ) adenocarcinoma. Ann. Oncol..

[30] Sahin U (2021). FAST: A randomised phase II study of zolbetuximab (IMAB362) plus EOX versus EOX alone for first-line treatment of advanced CLDN18.2-positive gastric and gastro-oesophageal adenocarcinoma. Ann. Oncol..

[31] Shah MA (2017). Effect of fluorouracil, leucovorin, and oxaliplatin with or without onartuzumab in HER2-negative, MET-positive gastroesophageal adenocarcinoma: The METGastric randomized clinical trial. JAMA Oncol.

[32] Catenacci DVT (2017). Rilotumumab plus epirubicin, cisplatin, and capecitabine as first-line therapy in advanced MET-positive gastric or gastro-oesophageal junction cancer (RILOMET-1): A randomised, double-blind, placebo-controlled, phase 3 trial. Lancet Oncol..

[33] Shitara K (2020). Efficacy and safety of pembrolizumab or pembrolizumab plus chemotherapy vs chemotherapy alone for patients with first-line, advanced gastric cancer: The KEYNOTE-062 phase 3 randomized clinical trial. JAMA Oncol..

[34] Kato K (2020). Pembrolizumab plus chemotherapy versus chemotherapy as first-line therapy in patients with advanced esophageal cancer: The phase 3 KEYNOTE-590 study. Ann. Oncol..

[35] Daniel VC (2017). KEYNOTE-059 cohort 3: Safety and efficacy of pembrolizumab monotherapy for first-line treatment of patients (pts) with PD-L1-positive advanced gastric/gastroesophageal (G/GEJ) cancer. Ann. Oncol..

[36] Chau I (2017). Ramucirumab (R) plus pembrolizumab (P) in treatment naive and previously treated advanced gastric or gastroesophageal junction (G/GEJ) adenocarcinoma: A multi-disease phase I study. J. Clin. Oncol..

[37] Yoshikawa T (2019). Effect of first-line S-1 plus oxaliplatin with or without ramucirumab followed by paclitaxel plus ramucirumab on advanced gastric cancer in East Asia: The phase 2 RAINSTORM Randomized Clinical Trial. JAMA Netw. Open.

[38] Malka D (2019). FOLFOX alone or combined with rilotumumab or panitumumab as first-line treatment for patients with advanced gastroesophageal adenocarcinoma (PRODIGE 17-ACCORD 20-MEGA): A randomised, open-label, three-arm phase II trial. Eur. J. Cancer.

[39] Fuchs CS (2019). Ramucirumab with cisplatin and fluoropyrimidine as first-line therapy in patients with metastatic gastric or junctional adenocarcinoma (RAINFALL): A double-blind, randomised, placebo-controlled, phase 3 trial. Lancet Oncol.

[40] Cleary JM (2019). FOLFOX plus ziv-aflibercept or placebo in first-line metastatic esophagogastric adenocarcinoma: A double-blind, randomized, multicenter phase 2 trial. Cancer.

[41] Bang YJ (2019). A phase II, randomised study of mFOLFOX6 with or without the Akt inhibitor ipatasertib in patients with locally advanced or metastatic gastric or gastroesophageal junction cancer. Eur. J. Cancer.

[42] Yoon HH (2016). Ramucirumab combined with FOLFOX as front-line therapy for advanced esophageal, gastroesophageal junction, or gastric adenocarcinoma: A randomized, double-blind, multicenter Phase II trial. Ann. Oncol..

[43] Tebbutt NC (2016). Panitumumab added to docetaxel, cisplatin and fluoropyrimidine in oesophagogastric cancer: ATTAX3 phase II trial. Br. J. Cancer.

[44] Shah MA (2016). A randomized phase II study of FOLFOX with or without the MET inhibitor onartuzumab in advanced adenocarcinoma of the stomach and gastroesophageal junction. Oncologist.

[45] Shen L (2015). Bevacizumab plus capecitabine and cisplatin in Chinese patients with inoperable locally advanced or metastatic gastric or gastroesophageal junction cancer: Randomized, double-blind, phase III study (AVATAR study). Gastric Cancer.

[46] Du F (2015). S-1 and cisplatin with or without nimotuzumab for patients with untreated unresectable or metastatic gastric cancer a randomized, open-label phase 2 trial. Medicine.

[47] Zhang ZD (2014). Clinical evaluation of cetuximab combined with an S-1 and oxaliplatin regimen for Chinese patients with advanced gastric cancer. World J. Surg. Oncol..

[48] Iveson T (2014). Rilotumumab in combination with epirubicin, cisplatin, and capecitabine as first-line treatment for gastric or oesophagogastric junction adenocarcinoma: An open-label, dose de-escalation phase 1b study and a double-blind, randomised phase 2 study. Lancet Oncol..

[49] Waddell T (2013). Epirubicin, oxaliplatin, and capecitabine with or without panitumumab for patients with previously untreated advanced oesophagogastric cancer (REAL3): A randomised, open-label phase 3 trial. Lancet Oncol..

[50] Lordick F (2013). Capecitabine and cisplatin with or without cetuximab for patients with previously untreated advanced gastric cancer (EXPAND): A randomised, open-label phase 3 trial. Lancet Oncol..

[51] Eatock MM (2013). Phase II randomized, double-blind, placebo-controlled study of AMG 386 (trebananib) in combination with cisplatin and capecitabine in patients with metastatic gastro-oesophageal cancer. Ann. Oncol..

[52] Ohtsu A (2011). Bevacizumab in combination with chemotherapy as first-line therapy in advanced gastric cancer: A randomized, double-blind, placebo-controlled phase III study. J. Clin. Oncol..

[53] Rao S (2010). Matuzumab plus epirubicin, cisplatin and capecitabine (ECX) compared with epirubicin, cisplatin and capecitabine alone as first-line treatment in patients with advanced oesophago-gastric cancer: A randomised, multicentre open-label phase II study. Ann Oncol.

