# Supplementary material for: Comparative efficacy and tolerability of targeted and immunotherapy combined with chemotherapy as first-line treatment for advanced gastric cancer: a Bayesian network meta-analysis
Source: Sci Rep. 2022 Dec 20;12:22024. doi: 10.1038/s41598-022-24426-9 (PMC9768117; doi:10.1038/s41598-022-24426-9)
Supplement: Supplementary file 1 — Supplementary Information. [file 41598_2022_24426_MOESM1_ESM.docx]

**Supplementary Materials**

Supplement Table S1: Search strategies: Page **1**

Supplement Table S2: Studies eligible for exclusion: Page **2**

Supplement Table S3: Risk of bias assessment of Average group’s studies: Page **3-7**

Supplement Table S4: Risk of bias assessment of Specific positivity group’s studies: Page **8-10**

Supplement Figure S1: Network plots of secondary outcomes in Average group: Page **13**

Supplement Figure S2: Network plots of secondary outcomes in Specific positivity group: Page **14**

Supplement Figure S3: Funnel plots for primary outcomes: Page **15**

Supplement Figure S4: Funnel plots for secondary outcomes: Page **16**

Supplement Figure S5: Network forest-plots for secondary outcomes (Average group): Page **17**

Supplement Figure S6: Network forest-plots for secondary outcomes (Specific positivity group): Page **18**

Supplement Figure S7: Network league table for secondary outcomes: Page **19**

Supplement Table S5: *I^2^* and DIC differences: Page **20**

References: page **20**

**Supplement Table S1: Search strategies**

1. PUBMED

#1 ((gastric cancer OR gastric carcinoma OR stomach cancer OR stomach neoplasm* OR stomach carcinoma OR gastric tumor OR stomach tumor OR esophagogastric cancer OR esophagogastric carcinoma OR esophagogastric neoplasm*) OR (“Stomach Neoplasms” [Mesh])) 164,658

#2 ((targeted therapy OR targeted-therapy OR immunotherapy OR chemotherapy OR chemo-therapy OR (“immunotherapy” [Mesh]) OR (“Molecular Targeted Therapy” [Mesh]) OR (“Immune Checkpoint Inhibitors” [Mesh])) 4,165,217

#3 (late OR metastatic OR “locally advanced” OR advanced OR inoperable OR unresected) 2,632,115

#4 (First line OR First-line OR untreated) 416,922

#1 AND #2 AND #3 AND #4 **items found 2040**

1. EMBASE

#1 gastric OR 'stomach'/exp OR stomach OR esophagogastric OR oesophagogastric OR gastroesophageal OR gastrooesophageal 697,829

#2 cancer OR carcinoma OR adenocarcinoma OR neoplasm OR 'neoplasm'/exp 5,240,507

#3 ‘targeted therapy’ OR immunotherapy OR chemotherapy OR ‘Immune Checkpoint Inhibitors’ OR ‘Molecular Targeted Therapy’ 1,243,713

#4 late OR metastatic OR 'locally advanced' OR advanced OR inoperable OR unresected 1,961,943

#5 First line OR First-line OR untreated 604,089

#1 AND #2 AND #3 AND #4 AND #5 **items found** **3182**

1. Cochrane library

#1 gastric OR stomach OR esophagogastric OR oesophagogastric OR gastroesophageal OR gastrooesophageal 38,397

#2 cancer OR carcinoma OR adenocarcinoma OR neoplasm 212,576

#3 targeted therapy OR immunotherapy OR chemotherapy OR chemo-therapy OR targeted-therapy OR Molecular Targeted Therapy OR Immune Checkpoint Inhibitors 135,422

#4 late OR metastatic OR locally advanced OR advanced OR inoperable OR unresected 122,509

#5 first line OR first-line OR untreated 46,500

#6 #1 AND #2 AND #3 AND #4 AND #5 **items found 904**

**Supplement Table S2: Studies eligible for exclusion**

| Study | Reason for exclusion |
| --- | --- |
| Li 2020^1^ | Chemotherapy (Tegafur) unqualified with NCCN 2.2021 guideline |
| Boku 2019^2^ | Could not incorporate into network calculation (SOX plus nivolumab vs XELOX plus nivolumab) |
| Koizumi 2013^3^ | Recurrence patients previously received systemic chemotherapy |
| Richard 2013 ^4^ | Patients previously received 5-fluorouracil and leucovorin |

**Supplement Table S3: Risk of bias assessment of Average group’s studies**

| Study | Random sequence generation | Allocation concealment | Blinding of participants and personnel | Blinding of outcome assessment | Incomplete outcome data | Selective reporting | Other Sources of bias |
| --- | --- | --- | --- | --- | --- | --- | --- |
| Shan 2021 | Unclear, no specific description | Unclear, no specific description | High, open label design | Unclear, no specific description | Unclear, no specific description | Low, all expected endpoints have been reported | Unclear, no specific description |
| Shah 2021 | Low, permuted block randomization | Low, via interactive web response system | Low, Double blind placebo-controlled trial | Low, an independent data monitoring committee (DMC) reviewed | Low, OS, PFS, ORR and AE reported number close to total random patients in each arm | Low, all expected endpoints have been reported | Low, baseline  characteristics were well  balanced. |
| Boku 2020 | Unclear, no specific description | Unclear, no specific description | Low, Double blind placebo-controlled trial | Unclear, no specific description | Unclear, no specific description | Low, all expected endpoints have been reported | Unclear, no specific description |
| Kato 2020 | Unclear, no specific description | Unclear, no specific description | Low, Double blind placebo-controlled trial | Unclear, no specific description | Unclear, no specific description | Low, all expected endpoints have been reported | Unclear, no specific description |
| Mochler 2020 | Low, permuted block randomization | Low, via interactive web response system | High, open label design | Low, an independent data monitoring committee reviewed | Low, OS, PFS, ORR and AE reported number close to total random patients in each arm | Low, all expected endpoints have been reported | Low, baseline  characteristics were well  balanced. |
| Yoshikawa 2019 | Low, permuted block randomization | Low, via interactive web response system allocation | Low, Double blind placebo-controlled trial | Low, independent data reviewed | Low, OS, PFS, ORR and AE reported number close to total random patients in each arm | Low, all expected endpoints have been reported | Low, baseline  characteristics were well  balanced. |
| Malka 2019 | Unclear, no specific description | Unclear, no specific description | High, open label design | Unclear, no specific description | Low, OS, PFS, ORR and AE reported number close to total random patients in each arm | Low, all expected endpoints have been reported | Low, baseline  characteristics were well  balanced. |
| Fuchs 2019 | Low, interactive web response system generation random sequence | Low, via interactive web response system allocation | Low, Double blind placebo-controlled trial | Unclear, no specific description | Low, OS, PFS, ORR and AE reported number close to total random patients in each arm | Low, all expected endpoints have been reported | Low, baseline  characteristics were well  balanced. |
| Cleary 2019 | Unclear, no specific description | Unclear, no specific description | Low, Double blind placebo-controlled trial | Unclear, no specific description | Low, OS, PFS, ORR and AE reported number close to total random patients in each arm | Low, all expected endpoints have been reported | Low, baseline  characteristics were well  balanced. |
| Bang 2019 | Unclear, no specific description | Unclear, no specific description | Low, Double blind placebo-controlled trial | Unclear, no specific description | Low, OS, PFS, ORR and AE reported number close to total random patients in each arm | Low, all expected endpoints have been reported | Low, baseline  characteristics were well  balanced. |
| Yoon 2016 | Unclear, no specific description | Unclear, no specific description | Low, Double blind placebo-controlled trial | Unclear, no specific description | Low, OS, PFS, ORR and AE reported number close to total random patients in each arm | Low, all expected endpoints have been reported | Low, baseline  characteristics were well  balanced. |
| Tebbutt 2016 | Low, minimization method | Unclear, no specific description | High, open label design | Unclear, no specific description | Low, OS, PFS, ORR and AE reported number close to total random patients in each arm | Low, all expected endpoints have been reported | Low, baseline  characteristics were well  balanced. |
| Shah 2016 | Unclear, no specific description | Low, via interactive web response system allocation | Low, Double blind placebo-controlled trial | Low, ORR was independent reviewed | Low, OS, PFS, ORR and AE reported number close to total random patients in each arm | Low, all expected endpoints have been reported | Low, baseline  characteristics were well  balanced. |
| Shen 2016 | Low, minimization method | Low, via interactive web response system allocation | Low, Double blind placebo-controlled trial | Low, ORR was reviewed by investigators | Low, OS, PFS, ORR and AE reported number close to total random patients in each arm | Low, all expected endpoints have been reported | Low, baseline  characteristics were well  balanced. |
| Du 2015 | Low, permuted block randomization | Low, via interactive web response system allocation | High, open-label design | Unclear, no specific description | Low, OS, PFS, ORR and AE reported number close to total random patients in each arm | Low, all expected endpoints have been reported | High, early terminated |
| Zhang 2014 | Unclear, no specific description | Unclear, no specific description | High, method of drug administration (i.v) can’t be blind | Unclear, no specific description | Low, OS, PFS, ORR and AE reported number close to total random patients in each arm | Low, all expected endpoints have been reported | Unclear, patient recruitment amount in two arms w not descript |
| Iveson 2014 | Low, permuted block randomization | Low, via interactive web response system allocation | Low, Double blind placebo-controlled trial | Low, ORR was reviewed by investigators | Low, OS, PFS, ORR and AE reported number close to total random patients in each arm | Low, all expected endpoints have been reported | Low, baseline  characteristics were well  balanced. |
| Waddell 2013 | Low, permuted block randomization | Low, Central allocation | High, open label design | Unclear, no specific description | Low, OS, PFS, ORR and AE reported number close to total random patients in each arm | Low, all expected endpoints have been reported | Low, baseline  characteristics were well  balanced. |
| Lordick 2013 | Low, permuted block randomization | Low, Central allocation | High, open label design | Low, data reviewed by independent committee | Low, OS, PFS, ORR and AE reported number close to total random patients in each arm | Low, all expected endpoints have been reported | Low, baseline  characteristics were well  balanced. |
| Eatock 2013 | Low, random sequence by computer | Low, via interactive web response system allocation | Low, Double blind placebo-controlled trial | Low, tumor response was reviewed by investigators | Low, PFS, ORR and AE reported number close to total random patients in each arm | Low, all expected endpoints have been reported | Low, baseline  characteristics were well  balanced. |
| Ohtsu 2011 | Low, permuted block randomization | Low, via interactive web response system allocation | Low, Double blind placebo-controlled trial | Unclear, no specific description | Low, OS, PFS, ORR and AE reported number close to total random patients in each arm | Low, all expected endpoints have been reported | Low, baseline  characteristics were well  balanced. |

**Supplement Table S4: Risk of bias assessment of Specific positivity group’s studies**

| Study | Random sequence generation | Allocation concealment | Blinding of participants and personnel | Blinding of outcome assessment | Incomplete outcome data | Selective reporting | Other Sources of bias |
| --- | --- | --- | --- | --- | --- | --- | --- |
| Janjigan 2021 | Unclear, no specific description | Unclear, no specific description | Low, Double blind placebo-controlled trial | Unclear, no specific description | Unclear, no specific description | Unclear, no specific description | Unclear, no specific description |
| Sahin 2021 | Unclear, no specific description | Unclear, no specific description | High, open-label design | Unclear, no specific description | Low, OS, PFS, ORR and AE reported number close to total random patients in each arm | Low, all expected endpoints have been reported | High, introduced new arm after randomized |
| Shitara 2020 | Low, random list generation | Low, allocated by central interactive voice response and integrated web response system | Low, Double blind placebo-controlled trial | High, assessed by sponsors | Low, OS, PFS, ORR and AE reported number close to total random patients in each arm | Low, all expected endpoints have been reported | Low, baseline  characteristics were well  balanced. |
| Kato 2020 | Unclear, no specific description | Unclear, no specific description | Low, Double blind placebo-controlled trial | Unclear, no specific description | Unclear, no specific description | Low, all expected endpoints have been reported | Unclear, no specific description |
| Mochler 2020 | Low, permuted block randomization | Low, via interactive web response system | High, open label design | Low, an independent data monitoring committee reviewed | Low, OS, PFS, ORR and AE reported number close to total random patients in each arm | Low, all expected endpoints have been reported | Low, baseline  characteristics were well  balanced. |
| Tabernero 2018 | Low, using permuted block randomisation system | Low, allocated by interactive voice or web response system | Low, double-blind placebo-controlled trial | Low, tumor response assessed by investigators | Low, OS, PFS, ORR and AE reported number close to total random patients in each arm | Low, all expected endpoints have been reported | Low, baseline  characteristics were well  balanced. |
| Shah 2017 | Low, using permuted block randomisation system | Unclear, no specific description | Low, double-blind placebo-controlled trial | Low, tumor response assessed by investigators | Low, OS, PFS, ORR and AE reported number close to total random patients in each arm | Low, all expected endpoints have been reported | High, early termination of patient recruitment |
| Catenacci 2017 | Low, using permuted block randomisation system | Low, allocated by interactive voice or web response system | Low, double-blind placebo-controlled trial | Unclear, no specific description | Low, OS, PFS, ORR and AE reported number close to total random patients in each arm | Low, all expected endpoints have been reported | Low, baseline  characteristics were well  balanced. |
| Schuler 2016 | Unclear, no specific description | Unclear, no specific description | High, open label design | Unclear, no specific description | Unclear, no specific description | Low, all expected endpoints have been reported | Unclear, no specific description |
| Hetch 2016 | Unclear, no specific description | Low, allocation by central system | Low, triple blind placebo-controlled trial | Low, triple blinded design | Low, OS, PFS, ORR and AE reported number close to total random patients in each arm | Low, all expected endpoints have been reported | Low, baseline  characteristics were well  balanced. |
| Rao 2010 | Low, randomization generated by computer | Low, allocated by interactive voice or web response system | High, open label design | Low, tumor response accessed by investigators | Low, OS, PFS, ORR and AE reported number close to total random patients in each arm | Low, all expected endpoints have been reported | Low, baseline  characteristics were well  balanced. |
| Bang 2010 | Low, using permuted block randomisation system | Low, allocated by interactive voice or web response system | High, open label design | Unclear, no specific description | Low, OS, PFS, ORR and AE reported number close to total random patients in each arm | Low, all expected endpoints have been reported | Low, baseline  characteristics were well  balanced. |

**Supplement Figure S1: Network plots of secondary outcomes in Average group**

**
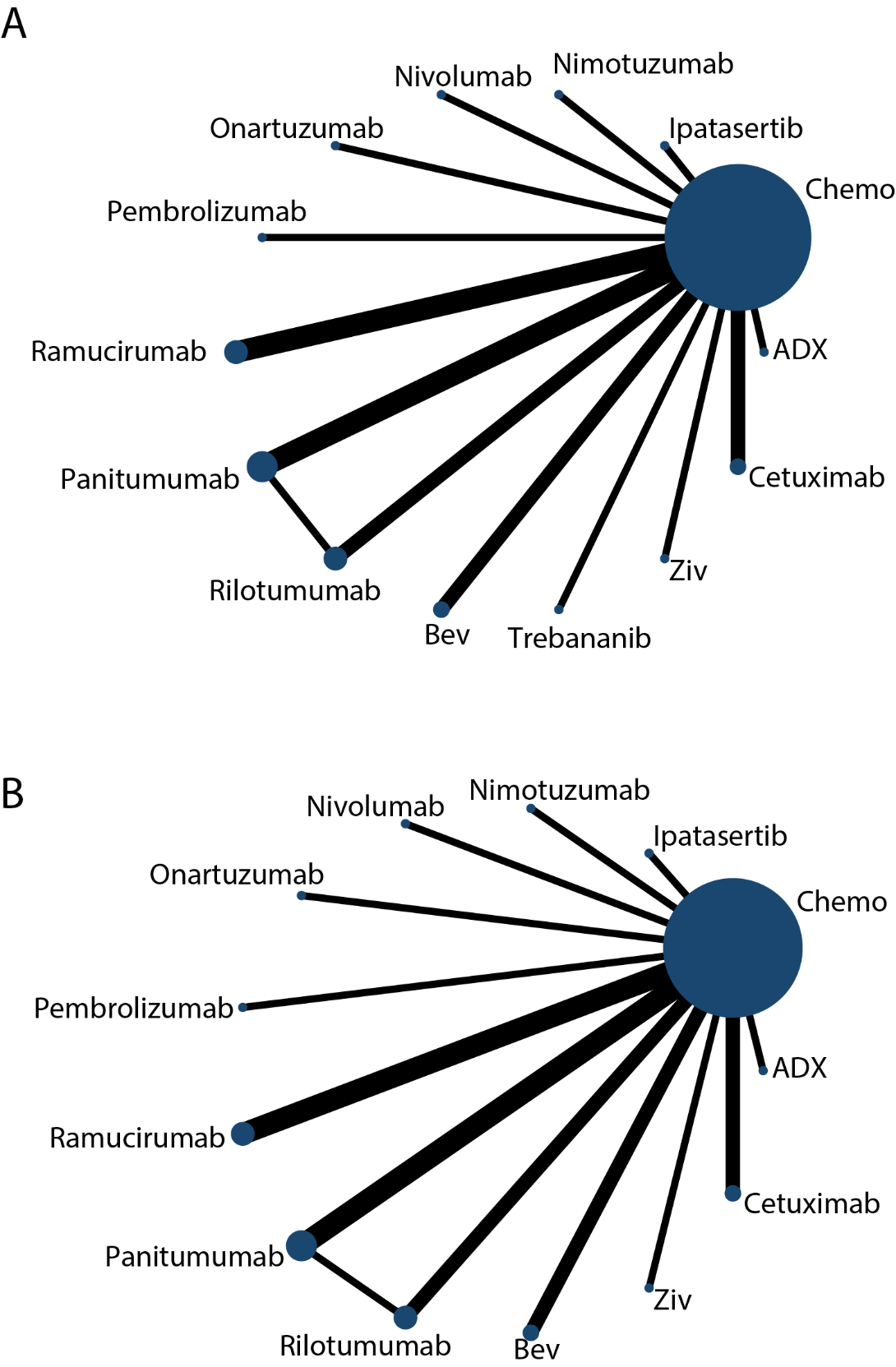
**

**A: Network plots of ORR (Average group) B: Network plots of AE≥3 (Average group)**

**Supplement Figure S2: Network plots of secondary outcomes in Specific positivity group**

**
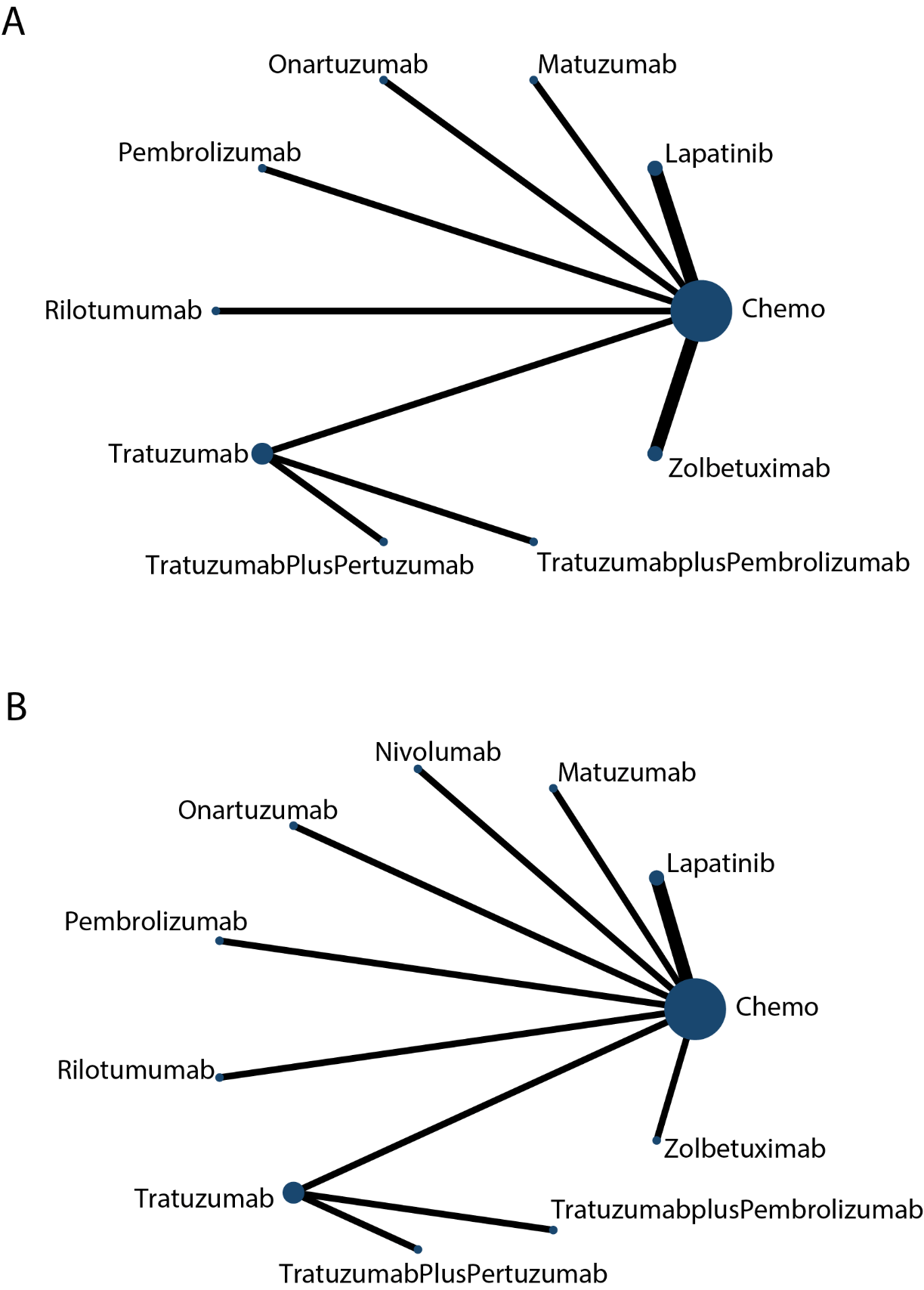
**

**A: Network plots of ORR (Specific positivity group); B: Network plots of AE≥3 (Specific positivity group)**

**Supplement Figure S3: Funnel plots for primary outcomes**


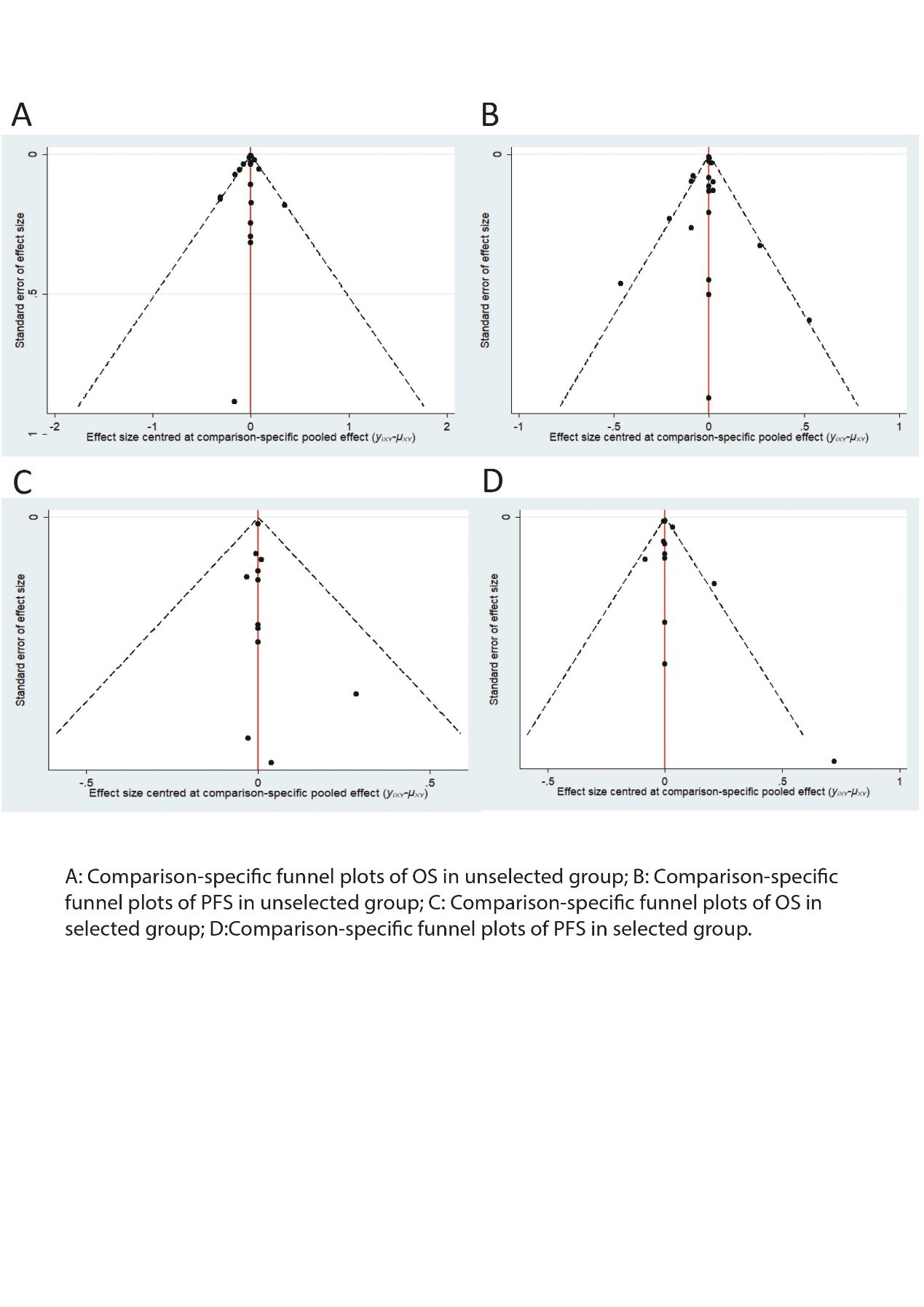


**A: Comparison specific funnel plots of OS in Average group; B: Comparison specific funnel plots of PFS in Average group; C: Comparison specific funnel plots of OS in Specific positivity group; D: Comparison specific funnel plots of PFS in Specific positivity group.**

**Supplement Figure S4: Funnel plots for secondary outcomes**

**
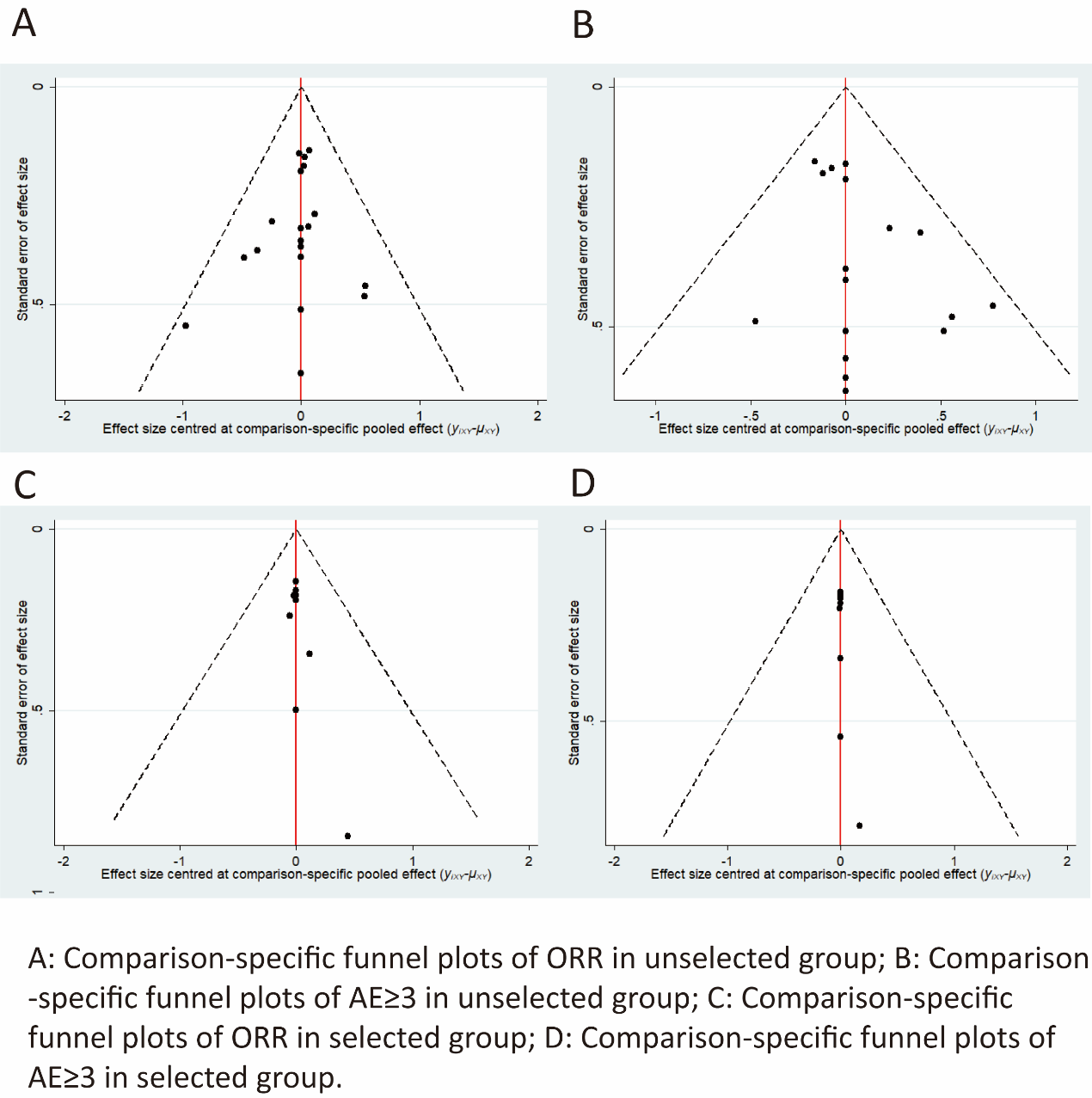
**

**A: Comparison specific funnel plots of ORR in Average group; B: Comparison specific funnel plots of AE≥3 in Average group; C: Comparison specific funnel plots of ORR in Specific positivity group; D: Comparison specific funnel plots of AE≥3 in Specific positivity group.**

**Supplement Figure S5: Network Forest plots for secondary outcomes (Average group)**

**
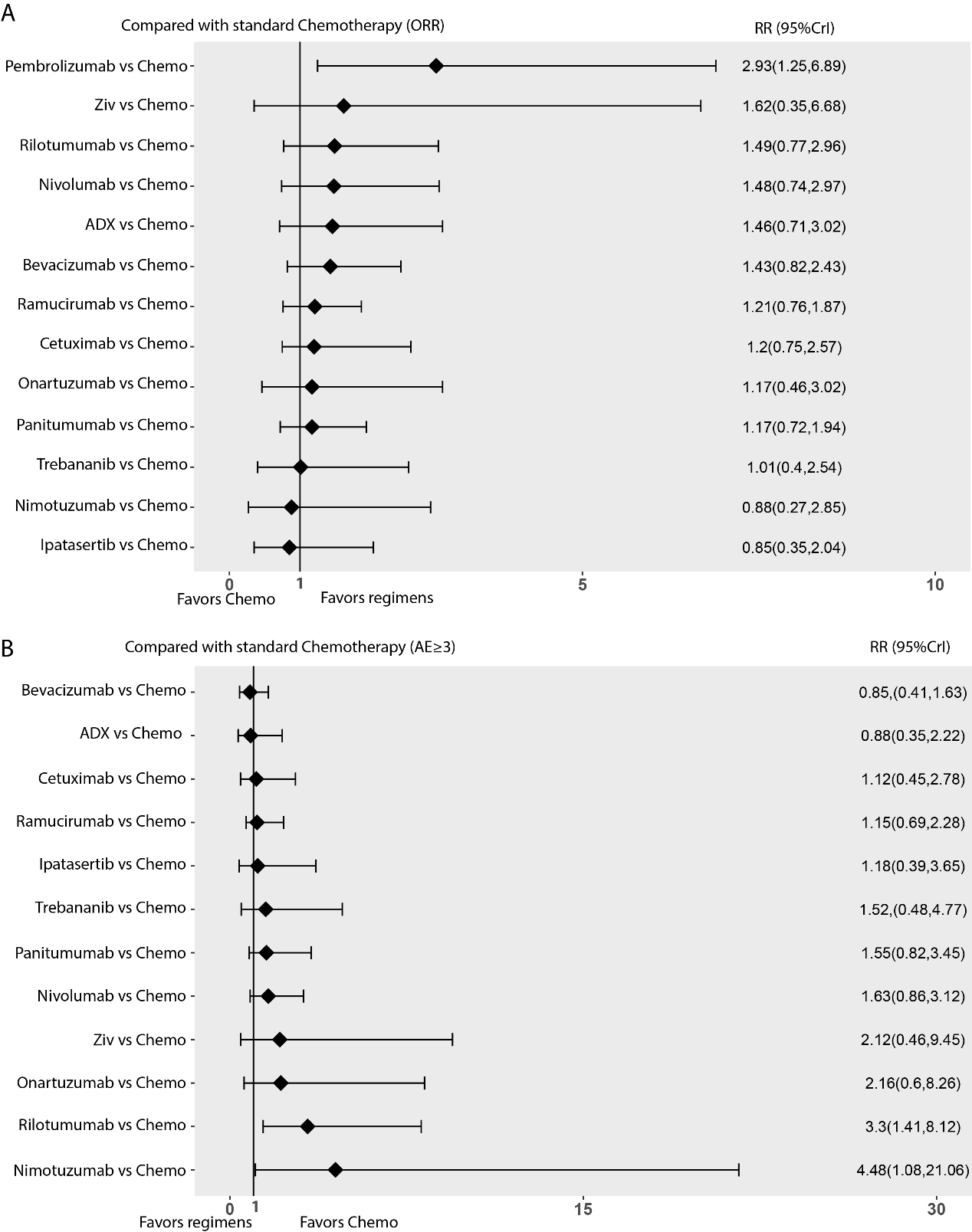
**

**A: The network forest plots vs chemotherapy of ORR in Average group; B: The network forest plots vs chemotherapy of AE≥3 in Average group**

**Supplement Figure S6: Network Forest plots for secondary outcomes (Specific positivity group)**


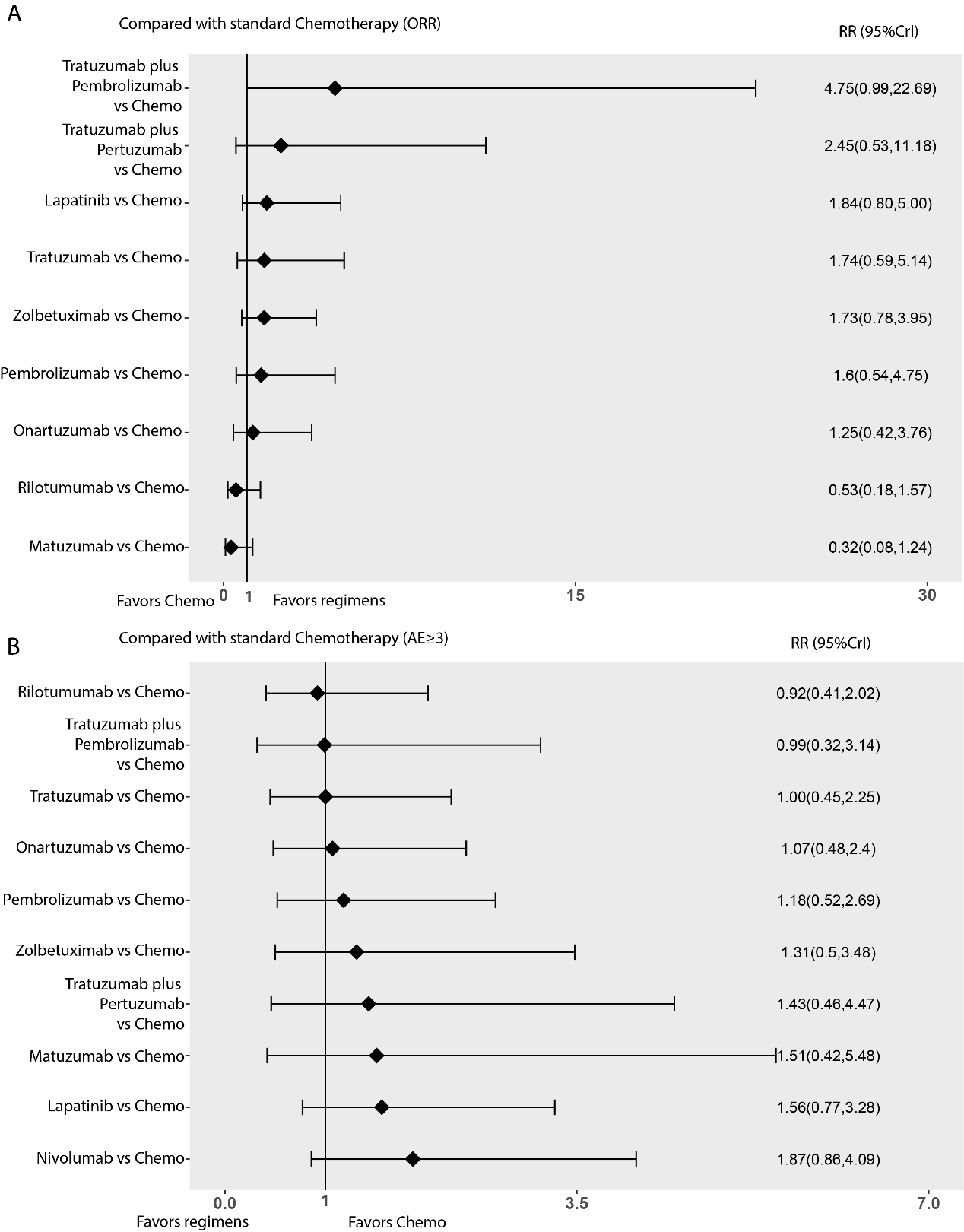


**A: The network forest plots vs chemotherapy of ORR in Specific positivity group; B: The network forest plots vs chemotherapy of AE≥3 in Specific positivity group**

**Supplement Figure S7:** **Network league table for secondary outcomes**

**
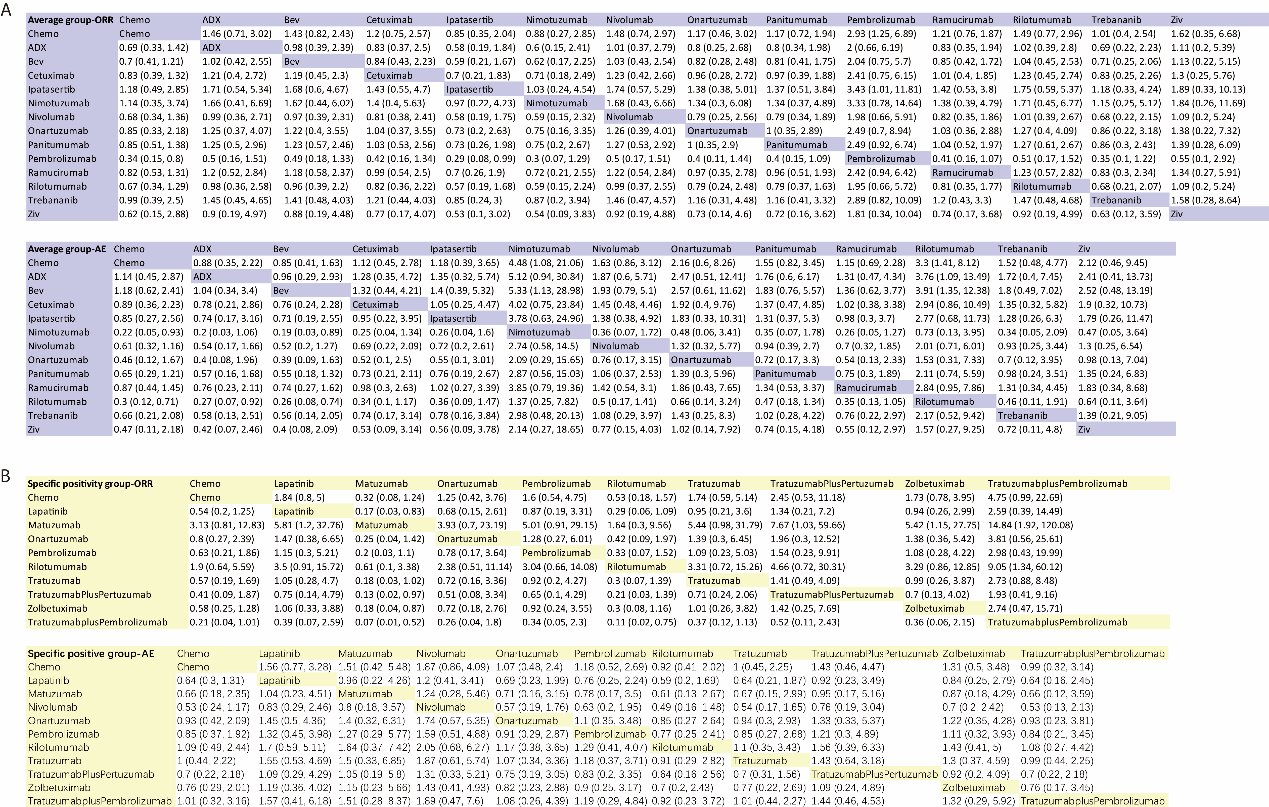
**

**A: The network league table for secondary outcomes in Average group; B: The network league table for secondary outcomes in Specific positivity group**

**Supplement Table S5: *I^2^* and DIC differences**

| **Average** | PFS | OS | ORR | AE |
| --- | --- | --- | --- | --- |
| *I^2^* | 6% | 5% | 4% | 5% |
| DIC consistency  DIC inconsistency | 40.023  40.105 | 35.973  35.995 | 76.611  76.328 | 68.613  68.498 |
| **Specific positivity** | PFS | OS | ORR | AE |
| *I^2^* | 5% | 0% | 0.5% | 2% |
| DIC consistency  DIC inconsistency | 22.435  22.501 | 19.975  19.828 | 41.976  41.868 | 42.692  42.720 |

**References**

1. Li C, Tang T and Wang W. Combination Use of Tegafur and Apatinib as First-Line Therapy in Treatment of Advanced Gastric Cancer: A Single-Blinded Randomized Study. Gastroenterol Res Pract 2020; 2020: 3232950. 2020/04/25. DOI: 10.1155/2020/3232950.

2. Boku N, Ryu MH, Kato K, et al. Safety and efficacy of nivolumab in combination with S-1/capecitabine plus oxaliplatin in patients with previously untreated, unresectable, advanced, or recurrent gastric/gastroesophageal junction cancer: interim results of a randomized, phase II trial (ATTRACTION-4). Annals of oncology : official journal of the european society for medical oncology 2019; 30: 250‐258. Clinical Trial, Phase II; Journal Article; Multicenter Study; Randomized Controlled Trial; Research Support, Non‐U.S. Gov't. DOI: 10.1093/annonc/mdy540.

3. Koizumi W, Yamaguchi K, Hosaka H, et al. Randomised phase II study of S-1/cisplatin plus TSU-68 vs S-1/cisplatin in patients with advanced gastric cancer. Brit J Cancer 2013; 109: 2079‐2086. Clinical Trial, Phase II; Journal Article; Multicenter Study; Randomized Controlled Trial; Research Support, Non‐U.S. Gov't. DOI: 10.1038/bjc.2013.555.

4. Richards D, Kocs DM, Spira AI, et al. Results of docetaxel plus oxaliplatin (DOCOX) ± cetuximab in patients with metastatic gastric and/or gastroesophageal junction adenocarcinoma: results of a randomised Phase 2 study. Eur J Cancer 2013; 49: 2823-2831. 2013/06/12. DOI: 10.1016/j.ejca.2013.04.022.
